# Nanoparticles targeting liver sinusoidal endothelial cells improve tolerance to vector and transgene antigens through tolerance spreading

**DOI:** 10.1016/j.ymthe.2026.03.009

**Published:** 2026-03-12

**Authors:** Romain Hardet, Shu-Hung Wang, Sandrine Delignat, Marine Blandin, Reinaldo Digigow, Cornelia Gottwick, Markus Heine, Lígia Margarida Marques Mesquita, Marco Fanzutti, Anna-Lisa Vocaturo, Disha Mungalpara, Olivier Boyer, Sébastien Lacroix-Desmazes, Sabine Fleischer, Sahil Adriouch

**Affiliations:** 1 University Rouen Normandie, INSERM, Normandie University, PANTHER, UMR 1234, 76000 Rouen, France; 2 Topas Therapeutics GmbH, Hamburg, Germany; 3 Institut National de la Santé et de la Recherche Médicale, Centre de Recherche des Cordeliers, CNRS, Sorbonne Université, Université de Paris, 75006 Paris, France; 4 Department of Medicine, University Medical Centre Hamburg-Eppendorf, Hamburg, Germany; 5 Department of Biochemistry and Molecular Cell Biology (N30), University Medical Clinic Hamburg-Eppendorf, Hamburg, Germany; 6 Department of Immunology and Biotherapy, CHU de Rouen, Rouen, France

**Keywords:** nanoparticles, liver sinusoidal endothelial cells, LSECs, immune tolerance, tolerance spreading, adeno-associated viral vectors, AAV, transgene persistence

## Abstract

Liver sinusoidal endothelial cells (LSECs) naturally cross-present antigens and induce T cell tolerance. Targeting LSECs with peptide-coupled nanoparticles offers an efficient strategy to induce antigen-specific immune tolerance. Previous preclinical and clinical studies have shown that peptide-coupled nanoparticles can effectively inhibit T cell responses to the selected cognate peptide epitopes. However, clinical situations such as viral-vector-mediated gene therapy would benefit from simultaneous tolerance induction to multiple epitopes/antigens, posing a significant challenge. In this study, we used mouse models of adeno-associated virus (AAV)-mediated gene transfer to assess the *in vivo* effects of peptide-loaded nanoparticles designed to tolerize immune responses to transgene- or/and capsid-derived epitopes. We report here for the first time that LSEC-targeting nanoparticles coupled to a single peptide epitope promoted extension of tolerance to multiple relevant epitopes/antigens and simultaneously tolerized both CD4^+^ and CD8^+^ T cell responses. This tolerance spreading, which we termed “Tspread,” remained specific to the transgene- and capsid-derived antigens conveyed by the administered AAV vector and did not impair immune responses to an unrelated vaccine antigen. These findings delineate a novel LSEC-mediated tolerance spreading with important clinical implications for viral-vector gene therapy, where tolerance to multiple epitopes/antigens is essential for long-term expression of therapeutic genes.

## Graphical abstract

**Figure:**
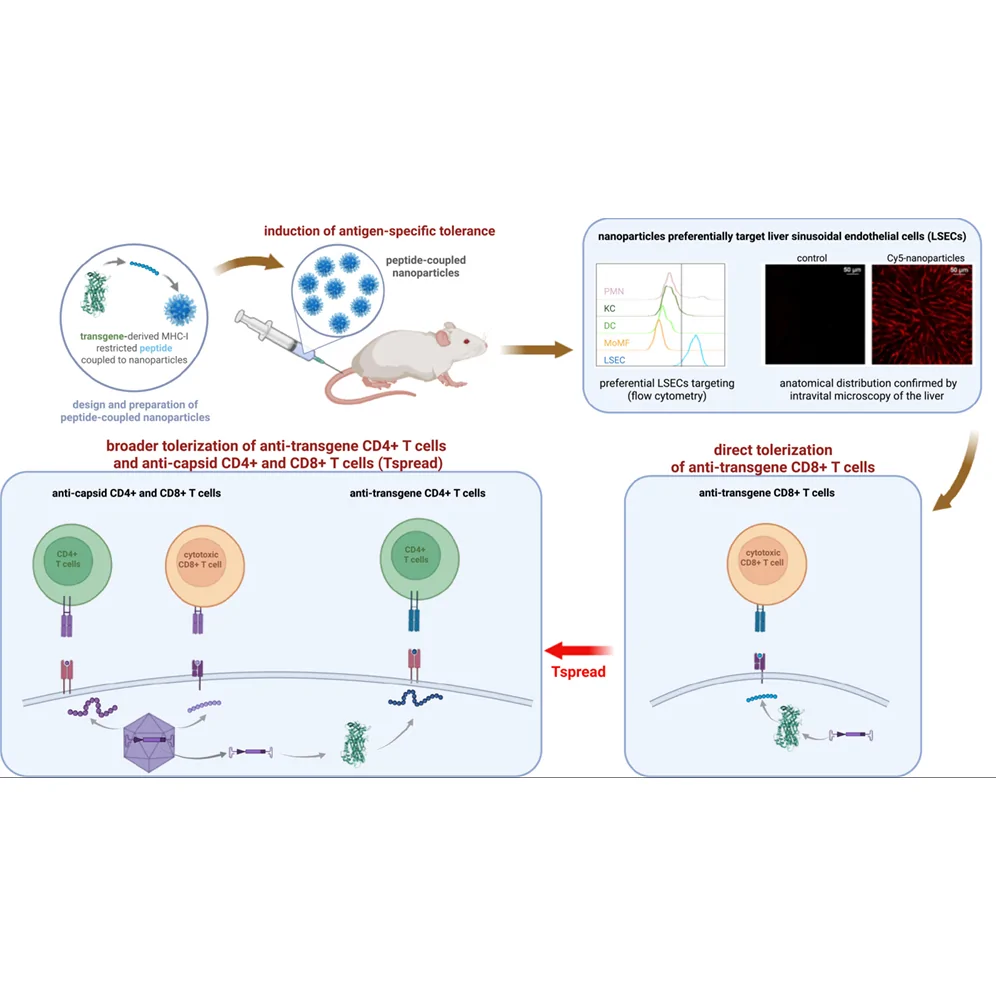

## Teaser

Anti-vector and anti-transgene immune responses represent a major bottleneck in AAV gene therapy. Peptide-coupled nanoparticles can deliver T cell epitopes to tolerogenic liver sinusoidal endothelial cells. Hardet and colleagues demonstrate that nanoparticles coupled to a single transgene-derived peptide induce broad tolerance toward transgene- and vector-specific CD4^+^ and CD8^+^ T cells through tolerance spreading (“Tspread”).

## Introduction

The liver is recognized as a highly tolerogenic organ containing various non-parenchymal liver cells (NPLCs), including unique conventional and unconventional antigen-presenting cell (APC) populations that finely modulate local and systemic tolerance and immunity.^1^ Among these NPLCs, liver sinusoidal endothelial cells (LSECs) stand out as unconventional APCs naturally exposed to gut-derived and blood-borne antigens, for which immune tolerance needs to be maintained. Equipped with remarkable clearance functions and the capacity to cross-present antigenic peptides,^2^ LSECs are considered the key mediators of immune tolerance owing to their unique potential to promote expansion of regulatory T cells (Tregs) and induce antigen-specific tolerization of CD4^+^ and CD8^+^ T cells by various mechanisms.^2^^,^^3^^,^^4^ Interestingly, unlike other tolerogenic APCs, LSECs can retain their tolerogenic functions even under moderate inflammatory conditions.^1^^,^^5^^,^^6^^,^^7^ Hence, leveraging their natural tolerogenic functions represents a promising strategy to favorably modulate immune responses *in vivo*.

Nanoparticles carrying peptides for tolerance induction, termed here tolerizing particle conjugates (TPCs), have been specifically designed to target LSECs. Indeed, upon their intravenous (i.v.) injection, TPCs have been shown to be preferentially taken up by LSECs and to effectively induce tolerance of the T cells specific for the peptide epitopes coupled to their surface.^8^^,^^9^ In several animal models of autoimmunity or immune dysregulation, treatment with TPCs coupled with disease-relevant peptides either prevented disease onset or significantly reduced its severity, being associated with Treg induction and inhibition of cognate CD8^+^ T cell immune responses.^8^^,^^9^^,^^10^ Recently, the proof of concept for antigen-specific tolerance induction was demonstrated in a clinical trial, where a mixture of TPCs coupled to a selected set of celiac-disease-relevant peptides was shown to tolerize T cell responses specific to these epitopes in patients (TCeD21 study, EudraCT number 2022-001656-41; ClinicalTrials.gov ID NCT05660109). However, in clinical situations such as viral vector gene transfer (GT), tolerance induction must target not only one or a few peptide epitopes but a multitude of different epitopes/antigens simultaneously, which is considered to represent a significant challenge.

GT using recombinant adeno-associated virus (AAV) vectors represents an effective therapeutic option for a variety of inherited monogenic diseases such as hemophilia.^11^^,^^12^^,^^13^ AAV vectors are recognized as relatively safe at moderate dose and efficient for achieving *in vivo* expression of therapeutic transgenic proteins.^14^^,^^15^ Depending on the AAV serotype, they can be used to transduce different target tissues with the objective of achieving long-term transgene expression.^13^^,^^16^^,^^17^^,^^18^ Yet, AAV-mediated GT triggers cellular and humoral immune responses directed against the viral capsid and the transgenic proteins, hindering long-term therapeutic efficacy.^19^^,^^20^^,^^21^^,^^22^ Indeed, cytotoxic CD8^+^ T cells can mediate the destruction of transduced cells and the loss of transgene expression, while humoral immunity can generate neutralizing antibodies against the capsid or the transgene product.^19^^,^^21^^,^^23^^,^^24^ Moreover, at high vector doses, both innate and adaptive immune responses contribute to vector toxicity, limiting the number of clinical situations suitable for AAV GT.^15^^,^^25^^,^^26^ To date, the main approaches to circumvent unwanted immune responses comprise the administration of immunosuppressive drugs and a strict selection of patients.^24^^,^^27^^,^^28^ Notwithstanding, immune responses can still arise despite immunosuppressive treatments and are accompanied by side effects that restrain their long-term administration.^15^^,^^28^^,^^29^ Hence, novel strategies to prevent or inhibit immune responses against the capsid and the transgenic protein are required to improve long-term transgene expression and to increase the eligibility to therapies based on AAV-mediated GT.^19^^,^^21^^,^^27^^,^^29^

To address this unmet medical need, we aimed in this study to leverage the robust tolerization potential of LSECs. To this end, we evaluated the capacity of TPCs to not only tolerize toward the selected cognate peptide epitopes coupled to the nanoparticles but also to extend the tolerance to other antigenic peptides derived from the AAV vectors and from the transgene. For this, TPCs coupled with selected capsid- or transgene-derived peptides were administered i.v. to mice to induce antigen-specific tolerization of T cells in the context of AAV-mediated muscle or liver-directed GT. As expected, our results showed that targeting LSECs using TPCs significantly inhibited CD8^+^ and CD4^+^ T cells directed against the targeted major histocompatibility complex class I (MHC-I)-restricted or MHC-II-restricted epitopes coupled to the nanoparticles. Strikingly, they also revealed a robust tolerance spreading toward other epitopes (i.e., not coupled to TPCs) carried by the same AAV vectors, resulting in a broader tolerization of both CD4^+^ and CD8^+^ T cells to multiple antigens. This tolerance spreading is reported here for the first time and was even observed when only a single MHC-I-restricted peptide epitope was coupled to TPCs, thereby demonstrating the potential of TPCs to induce tolerance spreading to capsid- and transgene-derived antigens in the context of AAV GT. Importantly, TPC-mediated tolerization to antigens derived from the vector or from the transgene remained restrained and did not impair the efficacy of simultaneous vaccination against an unrelated tumor antigen. Hence, exploiting the unique tolerization potential of LSECs via the precision of nanomedicine offers a novel approach to achieve tolerance to multiple antigens, notably in the context of vector-mediated GT.

## Results

### TPCs predominantly target LSECs in the liver

**Figure 1:**
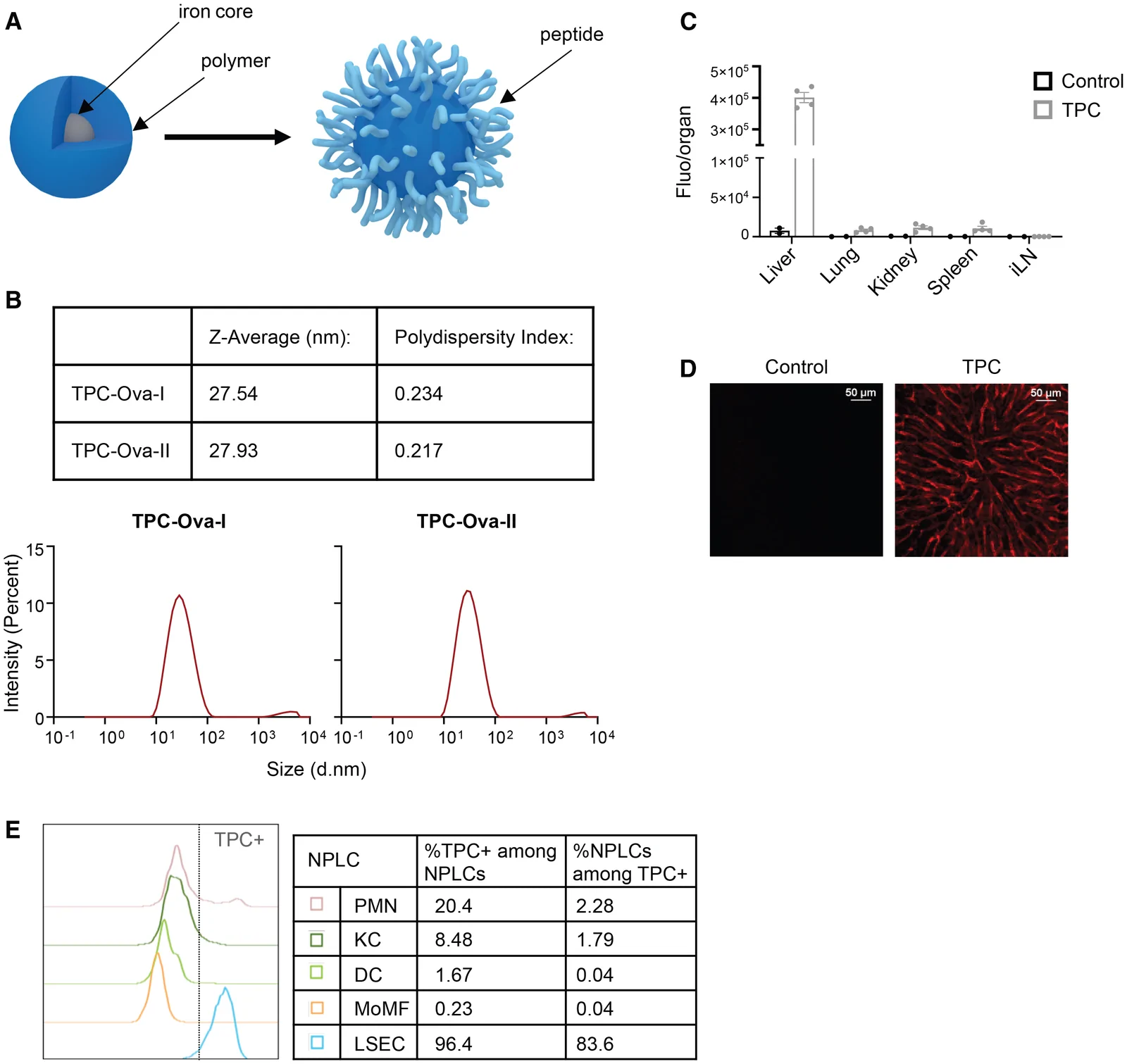
TPCs deliver peptides to the liver, predominantly to LSECs (A) Depiction of TPC nanoparticle composition. (B) Dynamic light scattering: size distribution of TPCs, average size (nm), and polydispersity index. (C) Biodistribution of TPCs *in vivo* in different organs. Ten minutes after i.v. administration of fluorescently labeled TPC-Ova-II-Cy5 (TPC-Cy5), the indicated organs and the inguinal lymph nodes (iLN) were collected, and the fluorescence intensity in the different organ lysates was analyzed. Each group constituted *n* = 4 animals. (D) Intravital microscopy of the liver was performed to evaluate the anatomical distribution of the florescence signal over time directly *in situ*. The presented illustrative pictures were taken 10 min after TPC-Cy5 i.v. injection, but similar data were also obtained at 15 min post injection. (E) Ten minutes after i.v. administration of TPC-Cy5, liver cells were isolated and analyzed by flow cytometry, whereby the NPLCs were defined as follows (after exclusion of the cell positives for CD3, CD19, or NK1.1 as shown in Figure S1): PMN (CD45^+^CD146^−^Ly6G^+^), KC (CD45^+^CD146^−^Ly6G^−^CD11b^+^F4/80^+^), DC (CD45^+^CD146^−^Ly6G^−^CD11b^int^CD11c^+^MHCII^+^), and MoMF (CD45^+^CD146^−^Ly6G^−^CD11b^int^CD11c^lo^MHCII^−^). The LSEC subset was defined as CD45^−^CD146^+^ (see also Figure S1 for the gating strategy). The representative histogram displays the fluorescence intensity of Cy5 signals for individual NPLC types (color coded). The percentage of TPC-Cy5^+^ cells in each indicated gated subset and the percentage of each subset among all TPC-Cy5^+^ cells are shown in the table. NPLC, non-parenchymal liver cells; PMN, polymorphonuclear neutrophils; KC, Kupffer cells; DC, dendritic cells; MoMF, monocyte-derived macrophages; LSEC, liver sinusoidal endothelial cells.

Tolerizing particles (TPs) comprise a superparamagnetic iron oxide core coated with a low-molecular-weight polymaleic acid-*alt*-octadecene polymer. TPs serve as a scaffold for conjugating selected peptides to their surface, resulting in tolerizing particle conjugates (TPCs), each carrying multiple copies of a single peptide (Figure 1A). In the first part of the present study, AAV-mediated GT in mice was conducted using a transgene encoding for the full-length secreted ovalbumin (Ova) protein, a known highly immunogenic secreted model antigen.^30^^,^^31^ To evaluate TPC-mediated tolerization, we generated TPCs, each coupled to one immunodominant peptide restricted by MHC classe I (Ova-I) or class II (Ova-II), corresponding to epitopes recognized by CD8^+^ and CD4^+^ T cells, respectively. These are referred to as TPC-Ova-I and TPC-Ova-II, which were also mixed in some experiments. Analytical characterization confirmed a uniform size distribution of both TPCs, averaging 25–30 nm, indicated by a low polydispersity index value (Figure 1B). To investigate the biodistribution of these nanoparticles, TPCs conjugated to Ova-II were fluorescently labeled with cyanine-5 (TPC-Ova-II-Cy5) and injected i.v. into wild-type mice. Ten minutes after injection, the liver displayed by far the highest fluorescence intensity among the organs assessed, whereas the spleen and kidneys remained at nearly background fluorescence levels (Figure 1C). Intravital microscopy of the liver further revealed rapid and preferential uptake of TPCs by LSECs, consistent with their high endocytic activity. Fluorescence signals were specifically detected along the liver sinusoidal lining, corresponding to the anatomical location of LSECs, with no visible staining of hepatocytes, Kupffer cells, or stellate cells (Figure 1D). Flow-cytometry analysis of NPLCs confirmed that LSECs represent the predominant cell population that captured TPC-Ova-II-Cy5 (>83%), with >96% of LSECs showing uptake 10 min after injection (Figure 1E).

### TPCs induce direct tolerization of cellular immune responses following AAV-mediated liver GT and improve transgene persistence

**Figure 2:**
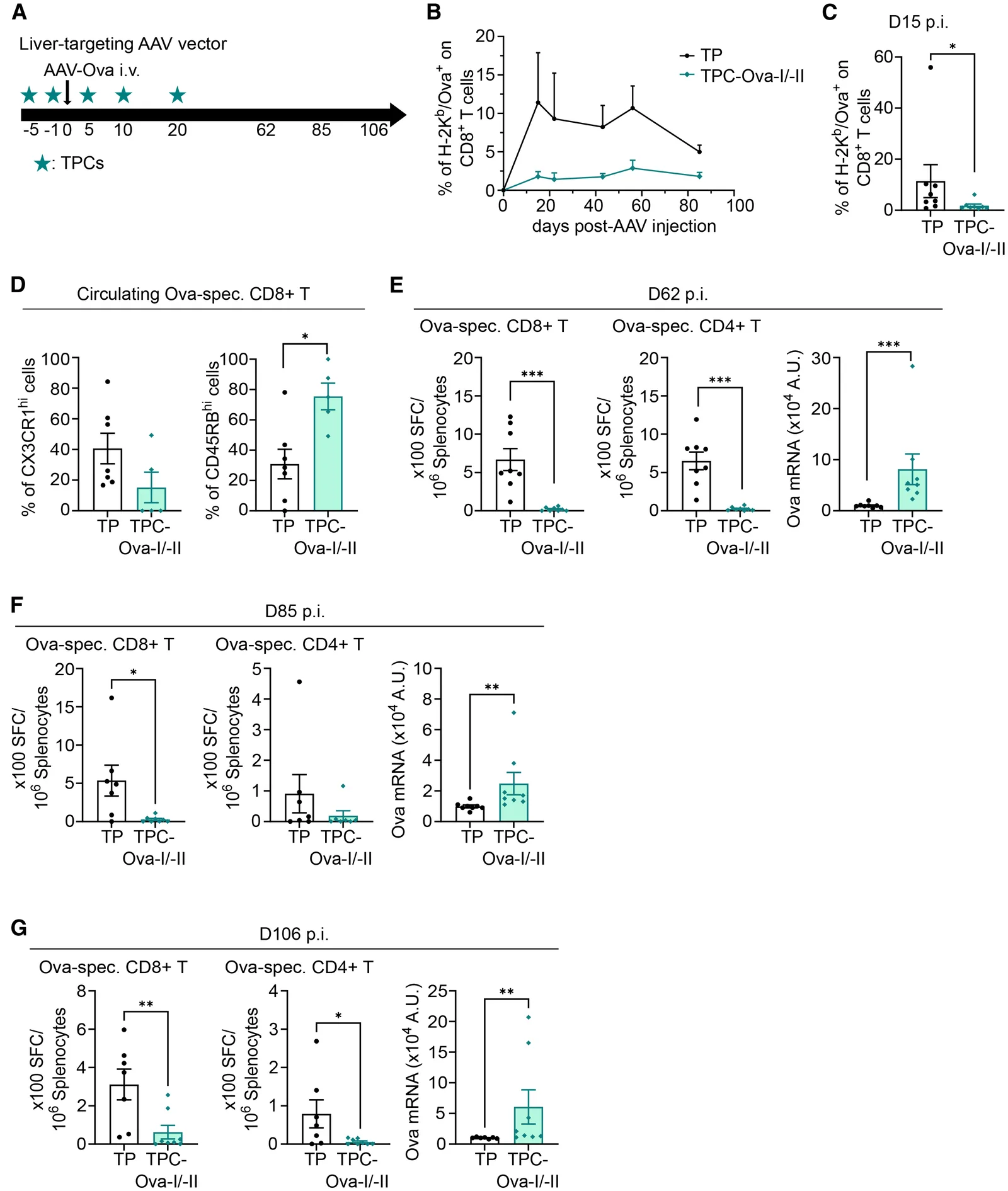
TPCs inhibit cellular immune responses induced by AAV-mediated liver gene transfer and improve transgene persistence (A) C57BL/6 mice were i.v. injected at day 0 with AAV8-Ova (2.5 × 10^11^ vg/kg) and received either empty particles as control (TPs) or a mixture of TPC-Ova-I and TPC-Ova-II (TPC-Ova-I/-II) i.v. at days −5, −1, 5, 10, and 20 post AAV injection (p.i.). The experiment was repeated with different study endpoints, at days 62, 85, or 106 p.i., as indicated. (B) Frequency over time of Ova-specific CD8^+^ T cells in the blood, evaluated by H-2K^b^/Ova-I dextramer staining. Animals received TPs (black circles) or TPC-Ova-I/-II (cyan diamonds). (C–G) Summary analysis comparing TP and TPC groups. Bars represent mean ± SEM. TPs, open bars; TPCs, closed bars. Group names are indicated on the *x* axis. (C) Frequency of Ova-specific CD8^+^ T cells in the blood at the peak of the response at day 15. (D) Frequency of CX3CR1^high^ cells and CD45RB^high^ cells on H-2K^b^/Ova-I^+^ CD8^+^ T cells in the blood at day 15. (E–G) Splenocytes were analyzed by ELISpot assays for their ability to secrete IFN-γ following *ex vivo* restimulation with MHC-I-restricted Ova-I and MHC-II-restricted Ova-II peptides. Transgene Ova mRNA in liver was assessed by RT-qPCR. The assays were performed at (E) day 62, (F) day 85, and (G) day 106, respectively. Each group constituted *n* = 8 animals. ∗*p* ≤ 0.05, ∗∗*p* ≤ 0.01 by Mann-Whitney tests for comparisons between TP and TPC-Ova-I/-II, and two-way ANOVA with Dunnett’s multiple comparisons test for kinetics.

We first evaluated the efficacy of TPCs to induce tolerization of the T cells that specifically recognize targeted specific epitopes (i.e., direct tolerization). In the context of AAV-mediated liver GT, we chose as a model a transgene coding for the highly immunogenic Ova with the aim of directly inhibiting Ova-specific T cells to tackle the deleterious anti-transgene cytotoxic T cells that eventually cause elimination of transduced cells.^30^^,^^31^ In this regard, mice were injected i.v. with AAV8-Ova at day 0 to transduce hepatocytes and received i.v. TPC-Ova-I (to induce tolerization of anti-Ova CD8^+^ T cells) and TPC-Ova-II (to induce tolerization of anti-Ova CD4^+^ T cells) at predefined time points, starting at day −5 and repeated four more times until day 20 post AAV injection (p.i.) (Figure 2A). Immune responses were monitored in blood samples from day 15 to the endpoint at day 106. The data in the control group show a prominent expansion of Ova-specific CD8^+^ T cells, which accounted for >10% of the CD8^+^ cells (Figure 2B). In contrast, animals treated with TPC-Ova-I/Ova-II displayed significantly lower levels of Ova-specific CD8^+^ T cells at all time points monitored (Figures 2B and 2C). The antigen-specific CD8^+^ T cells were not only dramatically reduced in TPC-tolerized mice, but the very few remaining cells also showed a less differentiated phenotype as indicated by the lower frequency of CX_3_CR_1_^hi^ cells and the higher frequencies of CD45RB^hi^ cells (a phenotype associated with low-affinity CD8^+^^32^^,^^33^^,^^34^), suggesting lower functional potency and affinity (Figure 2D). Moreover, analysis of their capability to secrete interferon-γ (IFN-γ) upon *ex vivo* peptide restimulation revealed an almost complete absence of Ova-I-reactive CD8^+^ as well as Ova-II-reactive CD4^+^ T cells (Figure 2E). In addition, higher transgene expression in the liver was observed in the tolerized group, suggesting a significant protection of hepatocytes from cytotoxic T cells *in vivo* (Figure 2E, right). Repetition of this experiment at different study endpoints similarly confirmed low numbers of Ova-I-reactive CD8^+^ T cells at days 85 and 106 p.i. and a progressive decline of Ova-II-reactive CD4^+^ T cells reaching near background level at day 106 p.i., along with significantly enhanced transgene persistence at all evaluated time points (Figures 2F and 2G).

### TPC-induced tolerization inhibits expansion of Ova-specific CD8^+^ T cells in the liver and promotes local activation of Tregs

**Figure 3:**
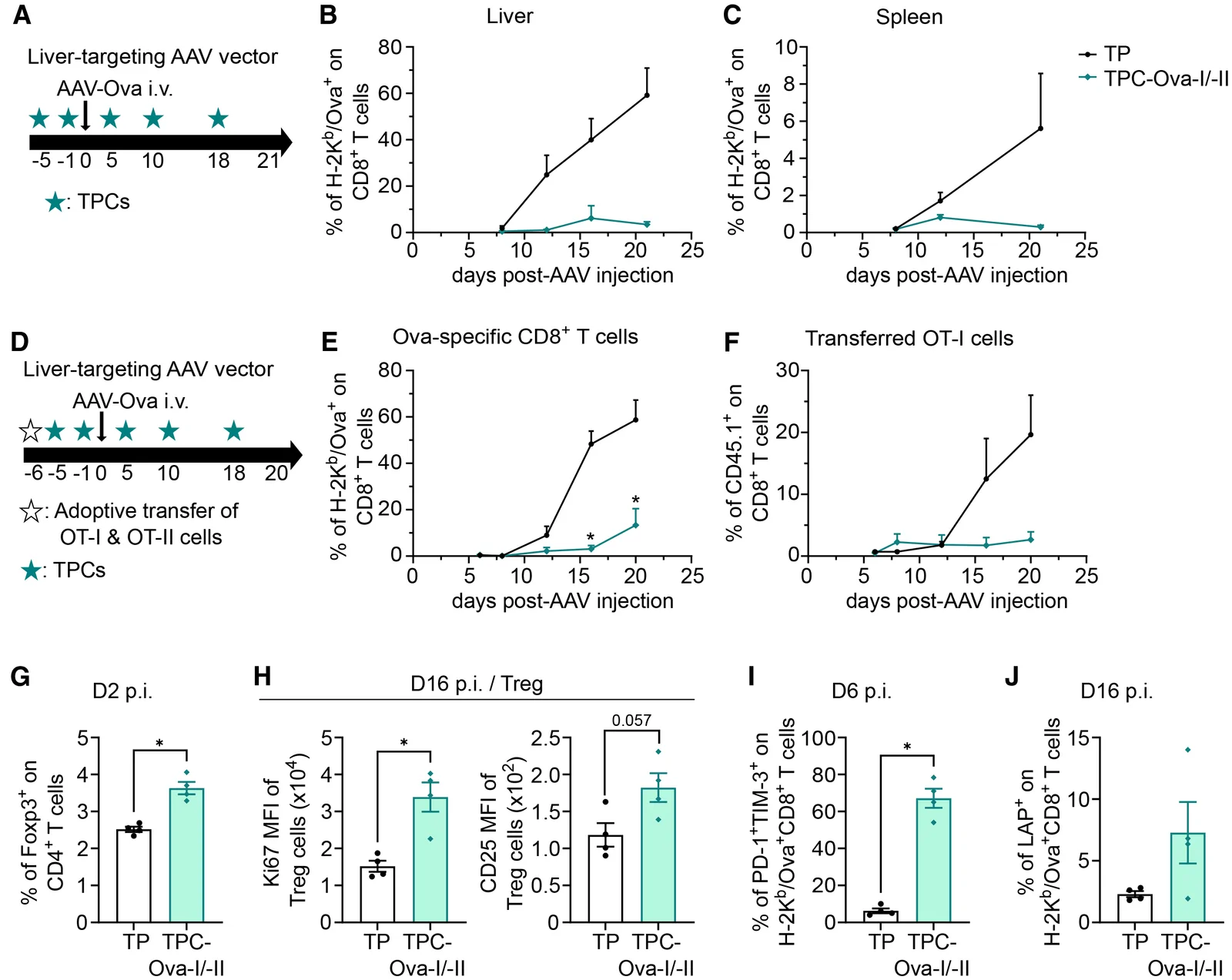
TPCs inhibit induction and expansion of Ova-specific CD8^+^ T cells and induce activation of Tregs in the liver (A) C57BL/6 mice were i.v. injected at day 0 with AAV8-Ova (5 × 10^11^ vg/kg) and received either empty particles as control (TPs) or TPC-Ova-I/-II i.v. at days −5, −1, 5, 10, and 18 post AAV injection (p.i.) (*n* = 16/group; at each time point, four mice were sacrificed). (B–C) Frequency of Ova-specific CD8^+^ T cells in the liver (B) and spleen (C) were determined by flow cytometry using H-2K^b^/Ova-I dextramer staining at the indicated time points after AAV injection. Animals received TPs (black circles) or TPC-Ova-I/-II (cyan diamonds). (D) C57BL/6 mice were i.v. injected at day 0 with AAV8-Ova (5 × 10^11^ vg/kg) and received TPC-Ova-I/-II i.v. at days −5, −1, 5, 10, and 18, post AAV injection (*n* = 20/group; at each time point, four mice were sacrificed). OT-I and OT-II cells were adoptively transferred into these animals 6 days before AAV injection. (E and F) Frequency of (E) total Ova-specific CD8^+^ T cells and (F) transferred OT-I TCR-transgenic CD8^+^CD45.1^+^ cells in the liver at the indicated time points. Grouping and visual encoding as in (B) and (C). (G–J) Summary analysis of additional immune parameters comparing TP with TPC groups. Bars represent mean ± SEM. TP, open bars; TPC, closed bars. Group names are indicated on the *x* axis. (G) Frequency of CD4^+^Foxp3^+^ Tregs in the liver at day 2. (H) Mean fluorescence intensity of Ki-67 and CD25 in CD4^+^Foxp3^+^ Tregs of the liver at day 16. (I) Frequency of Ova-specific CD8^+^ T cells expressing PD-1 and TIM-3 in the liver at day 6. (J) Frequency of Ova-specific CD8^+^ T cells expressing LAP in the liver at day 16. For each time point, *n* = 4 animals were analyzed. ∗*p* ≤ 0.05, ∗∗*p* ≤ 0.01 by Mann-Whitney test for comparisons between TP and TPC-Ova-I/-II, and two-way ANOVA with Dunnett’s multiple comparisons test for kinetics.

To investigate the mechanisms involved in TPC tolerization, we studied the priming and kinetics of immune responses in the targeted organ at early time points. As TPCs predominantly target LSECs (Figures 1C–1E), we studied the emergence of antigen-specific CD8^+^ T cells in the liver upon AAV8-Ova liver GT (Figure 3A). Control and TPC-tolerized animals were euthanized at different time points (i.e., 6, 12, 16, and 21 days p.i.), and the frequencies of antigen-specific CD8^+^ T cells were analyzed in the liver and spleen. In the control group, the results showed prominent priming and expansion of Ova-specific CD8^+^ T cells in the liver, becoming visible between days 6 and 12 p.i. and increasing to reach up to 60% of the total CD8^+^ T cells. In contrast, antigen-specific CD8^+^ T cells were barely detectable in the TPC-tolerized group before day 16 p.i. and remained at a low level throughout the study in both liver and spleen (Figures 3B and 3C).

To confirm the inhibition of antigen-specific T cells by TPC tolerization, we adoptively transferred T cell receptor (TCR)-transgenic Ova-specific T cells from OT-I and OT-II mice. CD8^+^ T cells from OT-I and CD4^+^ T cells from OT-II mice, both harboring the CD45.1 congenic marker, were mixed and adoptively transferred into CD45.2 recipient mice 1 day before the initiation of TPC tolerization (i.e., 6 days before AAV8-Ova injection) (Figure 3D). No expansion of the OT-II CD45.1^+^ CD4^+^ cells was observed at any time point (i.e., remaining below 0.5% of the total CD4^+^ T cells, data not shown). In contrast, antigen-specific CD8^+^ T cells were primed and expanded in non-tolerized mice between days 6 and 12 p.i., reaching about 60% of the total CD8^+^ T cells at the study endpoint (Figure 3E). In TPC-tolerized mice, endogenous antigen-specific CD8^+^ T cells remained significantly lower until study endpoint (Figure 3E). Priming and proliferation of adoptively transferred OT-I CD45.1^+^ cells remained also significantly low in this group, suggesting that even high-affinity TCR-transgenic CD8^+^ T cells are efficiently tolerized by TPC treatment (Figure 3F). Phenotypic analysis of liver T cells further revealed early activation of CD4^+^Foxp3^+^ Tregs, as suggested by their higher frequency at day 2 p.i. (Figure 3G) and their increased proliferation and activation status at day 16 p.i., as indicated, respectively, by a statistically significant elevated level of Ki-67 and an increased (albeit not reaching statistical significance) CD25 expression (Figure 3H). Concurrently, antigen-specific CD8^+^ cells significantly displayed a PD-1^+^TIM-3^+^ phenotype at day 6 p.i. (Figure 3I), which has been associated with terminally exhausted CD8^+^ T cells, and also exhibited a marked but not statistically significant increase in expression of latency-associated peptide (LAP) (Figure 3J), a marker expressed on different subsets of regulatory immune cells.^35^^,^^36^

### TPCs induce tolerization of T cells directed against the transgenic protein following AAV-mediated muscle GT

**Figure 4:**
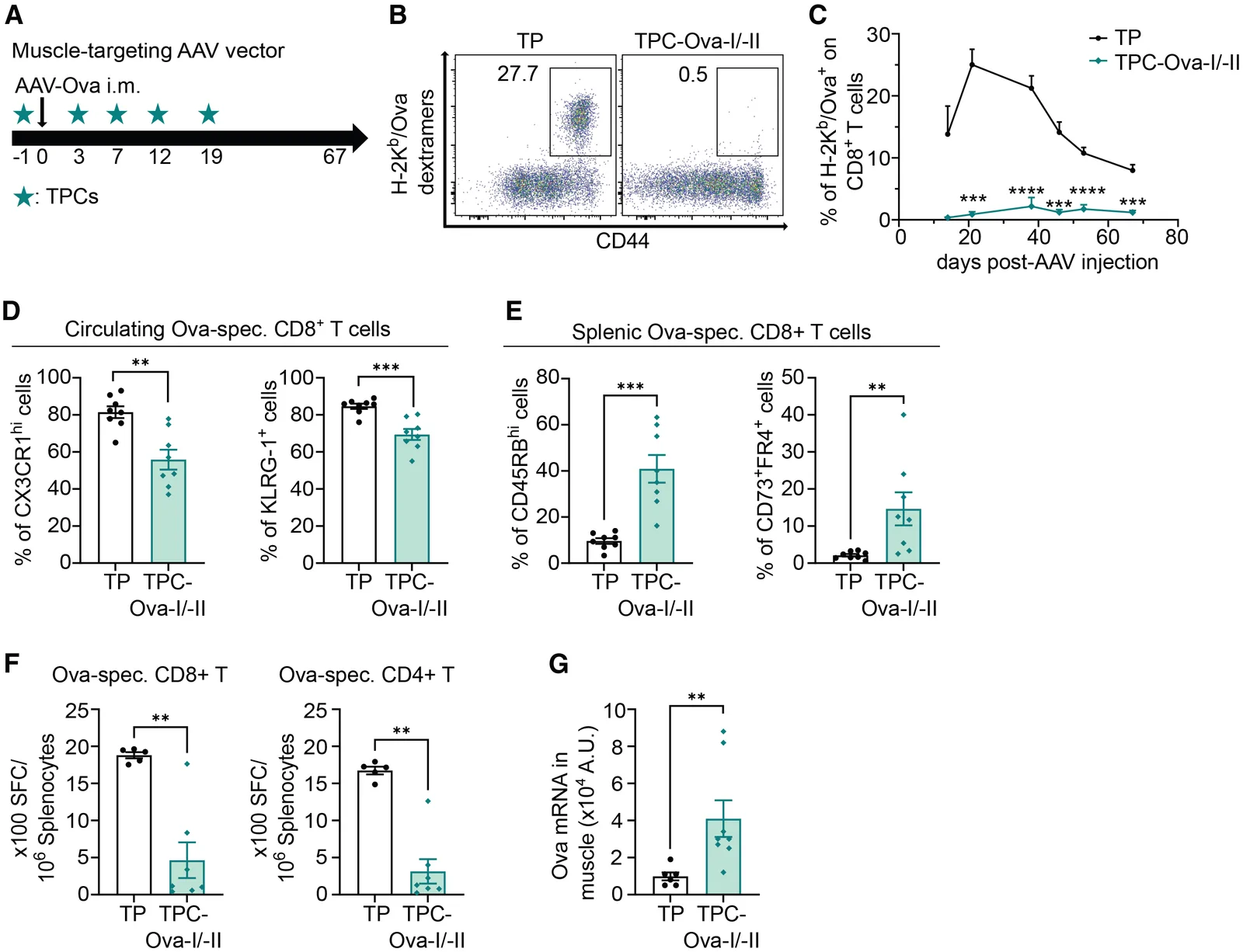
TPCs inhibit cellular immune responses induced by AAV-mediated muscle gene transfer and improve transgene persistence (A) C57BL/6 mice were i.m. injected at day 0 with AAV8-Ova (2.5 × 10^11^ vg/kg) in both gastrocnemii and received either empty particles as control (TPs) or TPC-Ova-I/-II injected i.v. at days −1, 3, 7, 12, and 19 post AAV injection (p.i.). (B and C) Frequency of circulating Ova-specific CD8^+^ T cells in the blood was determined by flow cytometry using H-2K^b^/Ova-I dextramer staining at the indicated time points after AAV injection. (B) Representative flow-cytometry panels. (C) Frequency of Ova-specific CD8^+^ T cells in the blood over time. Animals received TPs (black circles) or TPC-Ova-I/-II (cyan diamonds). (D–G) Summary analysis comparing TP and TPC groups. Bars represent mean ± SEM. TPs, open bars; TPCs, closed bars. Group names are indicated on the *x* axis. (D and E) Phenotyping of Ova-specific CD8^+^ T cells at day 67, (D) from blood samples (CX3CR1^hi^ and KLRG-1^+^ cells), and (E) from spleen samples (CD45RB^hi^ and CD73^+^FR4^+^ cells). (F) Splenocytes were also analyzed at day 67 by *ex vivo* ELISpot assays for their ability to secrete IFN-γ after restimulation with MHC-I-restricted Ova-I and MHC-II-restricted Ova-II peptides. (G) Transgene Ova mRNA in gastrocnemius muscles collected at day 67 were assessed by RT-qPCR. Each group constituted *n* = 8 animals. ∗*p* ≤ 0.05, ∗∗*p* ≤ 0.01, ∗∗∗*p* ≤ 0.001 by Mann-Whitney test for comparisons between TP and TPC-Ova-I/-II, and two-way ANOVA with Dunnett’s multiple comparisons test for kinetics.

As liver is considered an immune privileged environment, we then turned to the more immunogenic situation of AAV-mediated GT to the muscle, where robust anti-transgene CD8^+^ T cell responses have been reported.^37^^,^^38^ To evaluate the efficacy of TPCs to also induce tolerance in this other clinically relevant and more immunogenic situation, AAV8-Ova were injected intramuscularly (i.m.) at day 0, and mice received i.v. injection of an equimolar mixture of TPC-Ova-I and TPC-Ova-II at predefined time points, starting at day −1 and repeated four more times until day 19 p.i. (Figure 4A). Immune responses were monitored in blood samples from day 14 to the endpoint at day 67. In non-tolerized animals that received control TPs, up to 25% of circulating CD8^+^ T cells recognized Ova at the peak of the response at day 21 p.i., confirming a robust cytotoxic T cell response in this model. In contrast, antigen-specific CD8^+^ T cells remained significantly low in TPC-tolerized mice at all time points (Figures 4B and 4C). Again, the few Ova-specific CD8^+^ T cells that remained detectable in TPC-tolerized mice (Figure 4C) appeared to be less differentiated, as indicated by the lower frequency of CX_3_CR_1_^hi^ and KLRG-1^+^ cells at day 67 p.i. (Figure 4D).^33^^,^^34^^,^^39^ Moreover, in the spleen of TPC-tolerized animals, the remaining Ova-specific CD8^+^ T cells were significantly enriched in CD45RB^hi^ and CD73^+^FR4^+^ cells (Figure 4E), a phenotype associated with low-affinity CD8^+^ T cells^32^ and the acquisition of immunosuppressive functions,^40^^,^^41^ respectively.

### TPC-induced tolerization reduces the frequency of functional T cells and favors transgene long-term expression in the context of AAV-mediated muscle GT

To further investigate the functionality of antigen-specific T cells in TPC-tolerized mice, we used enzyme-linked immunospot (ELISpot) assays to assess their ability to secrete IFN-γ upon *ex vivo* restimulation with the immunodominant Ova-I and Ova-II peptides. ELISpot data showed a remarkable reduction in Ova-reacting CD4^+^ and CD8^+^ T cells in TPC-tolerized mice at day 67 p.i., confirming a significant inhibition of the expansion and function of antigen-specific T cells upon TPC tolerization (Figure 4F). Similar data were also obtained following AAV1-mediated Ova-transgene GT, suggesting TPC’s efficiency in inducing tolerance regardless of the AAV serotype (not shown). Moreover, we examined whether this correlated with an improved transgene persistence by quantifying the relative abundance of Ova mRNA in transduced muscles. Indeed, we observed higher transgene expression in the muscles of TPC-treated mice at day 67 p.i., confirming a significant positive impact on transgene persistence, also in this more immunogenic muscle site (Figure 4G), as demonstrated also in the previous experiments in the more tolerogenic liver site (Figures 2F and 2G).

### TPC treatment mediates tolerance spreading toward other relevant peptide epitopes carried by the same AAV vector

**Figure 5:**
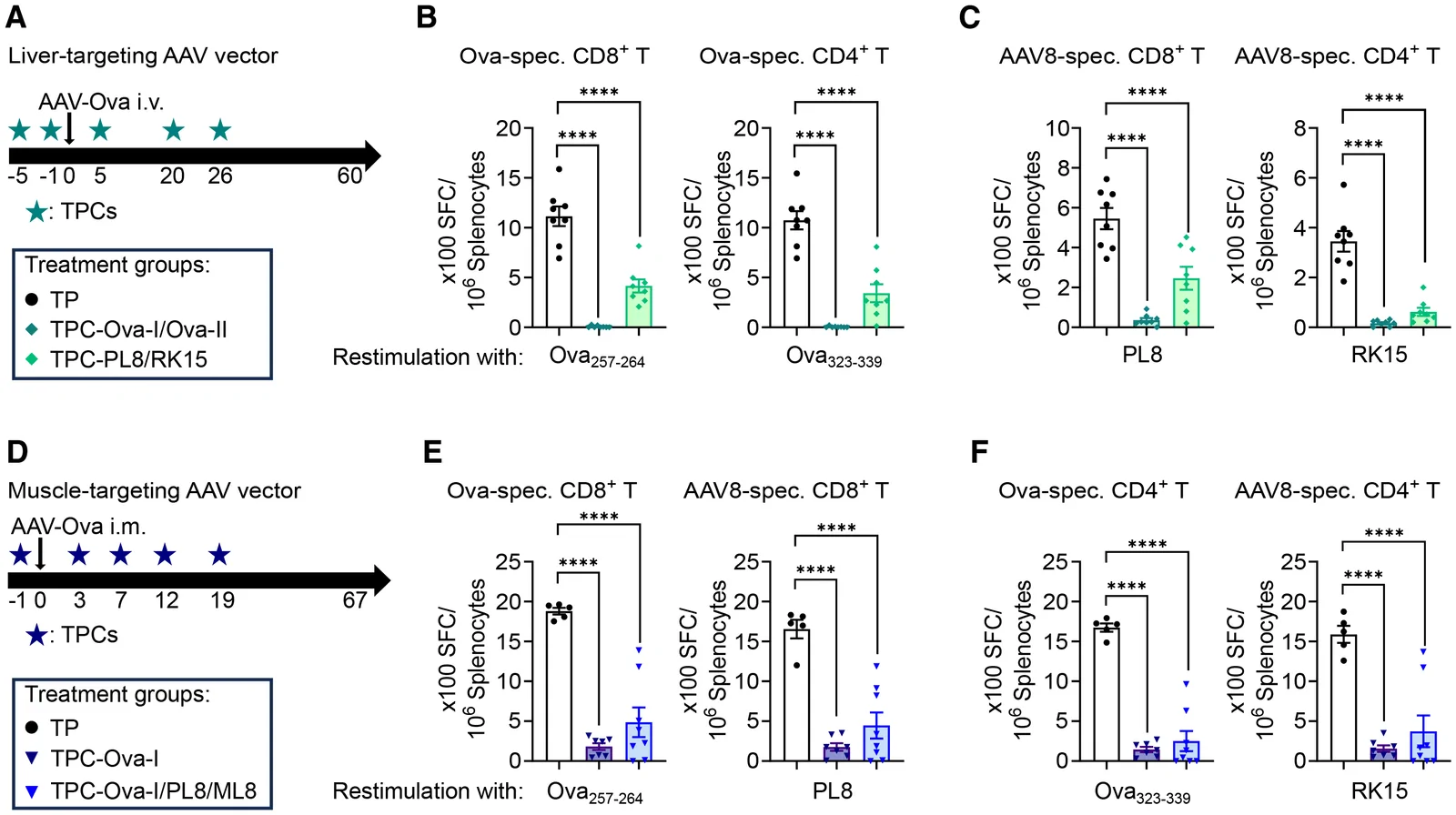
TPCs induce Tspread to other epitopes carried by the same AAV viral vector and between CD4^+^ and CD8^+^ T cell subpopulations (A) C57BL/6 mice were i.v. injected at day 0 with AAV8-Ova vector (2.5 × 10^11^ vg/kg) to transduce the liver and received TPs or TPCs coupled with both MHC-I- and MHC-II-restricted peptides derived either from the transgene (TPC-Ova-I/-II) or from the capsid (TPC-PL8/RK15) i.v. at days −5, −1, 5, 20, and 26 post AAV injection. Each group received the indicated treatment, consisting of i.v. injection of empty TPs (black circles), TPC-Ova-I/-II (dark-green diamonds), or TPC-PL8/RK15 (light-green diamonds). (B and C) Splenocytes were collected at day 60 and analyzed by ELISpot assays for their ability to secrete IFN-γ after restimulation with the indicated (B) MHC-I-restricted Ova_257–264_ or MHC-II-restricted Ova_323–339_ peptides, or (C) the indicated MHC-I-restricted capsid PL8 or MHC-II-restricted capsid RK15 peptides. The peptides used for *in vitro* restimulation are indicated on the *x* axis, and the corresponding specific T cell subsets expected to be activated by these peptides are indicated above each bar graph. (D) C57BL/6 mice were i.m. injected at day 0 with AAV8-Ova (2.5 × 10^11^ vg/kg) in both gastrocnemii and received TPs or TPCs coupled to MHC-I-restricted peptides derived either solely from the transgene (TPC-Ova-I) or from both transgene and capsid (a mixture of TPC-Ova-I/PL8/ML8) i.v. at days −1, 3, 7, 12, and 19 post AAV injection (p.i.). Each group received the indicated treatment, consisting of i.v. injection of empty TPs (black circles), TPC-Ova-I (purple triangles), or TPC-PL8/RK15 (blue triangles). (E and F) Splenocytes were collected at day 67 p.i. and analyzed by *ex vivo* ELISpot assays for their ability to secrete IFN-γ after restimulation with the indicated (E) MHC-I-restricted Ova_257–264_ or capsid PL8 peptides, or the indicated (F) MHC-II-restricted Ova_323–339_ or capsid RK15 peptides. The peptides used for *in vitro* restimulation are indicated on the *x* axis, and the corresponding specific T cell subsets expected to be activated by these peptides are indicated above each bar graph. Each group constituted *n* = 8 animals. Graphs show means ± SEM. ∗∗∗∗*p* ≤ 0.0001 by one-way ANOVA.

Having shown the efficiency of TPCs to tolerize T cells even in highly immunogenic situations, we then aimed to evaluate their effects more broadly on different relevant antigens. In particular, we investigated whether tolerization to a limited number of peptide epitopes (delivered to LSECs by TPCs) could potentially have a broader impact on other epitopes displayed in the same microenvironment (i.e., carried and/or expressed by the same AAV vector). To address this, we first tolerized the animals using a mixture of TPCs containing both MHC-I- and MHC-II-restricted peptides derived either only from the transgene (i.e., TPC-Ova-I/-II) or only from the AAV capsid (i.e., TPC-PL8/RK15) and evaluated the T cell responses to both transgene and capsid in the context of liver GT (Figure 5A). Strikingly, tolerization to transgene-derived epitopes also induced tolerance toward capsid-derived epitopes and, vice versa, tolerization to capsid-derived epitopes induced tolerance toward transgene-derived epitopes (Figures 5B and 5C). Subsequently, we tolerized the animals using only MHC-I-restricted peptides derived from the transgene and from the capsid (i.e., TPC-Ova-I/PL8/ML8) and injected AAV8-Ova (encoding as before the full-length ovalbumin protein) via the more immunogenic i.m. route (Figure 5D). We then evaluated the immune responses not only to the MHC-I-restricted peptides recognized by CD8^+^ T cells but also to the MHC-II-restricted peptides recognized by CD4^+^ T cells (Figures 5E and 5F). The data showed that TPCs loaded with only MHC-I-restricted epitopes (i.e., TPC-Ova-I/PL8/ML8) not only tolerized the CD8^+^ T cell responses (Figure 5E) but also the CD4^+^ T cell responses specific to both capsid- and transgene-derived epitopes (Figure 5F). Remarkably, the data also demonstrated that TPC-mediated tolerization toward a single transgene-derived epitope recognized by CD8^+^ T cells (i.e., using TPC-Ova-I), broadly tolerized CD4^+^ and CD8^+^ responses to both transgene and capsid epitopes, showing spreading of tolerance from one single CD8^+^ peptide epitope (i.e., which had been coupled to the TPCs) to multiple other antigens encoded by the vector. Collectively, these data indicate that the phenomenon of tolerance spreading (termed Tspread) observed here promotes extension of tolerance beyond the TPC-loaded epitopes, toward additional relevant antigenic epitopes displayed in the same microenvironment, and other T cell subsets.

### TPC treatment mediates Tspread in the context of a clinically relevant transgene carried by the same AAV vector

To assess whether the TPC-induced Tspread is not a specificity of the Ova model, we next turned to another more clinically relevant antigen. For this, we used an AAV vector coding for coagulation factor VIII (FVIII), reported to be very immunogenic in the context of FVIII-deficient mice, and to induce the priming of CD4^+^ T cells that further shape the humoral and cellular immune responses.^42^^,^^43^^,^^44^^,^^45^

**Figure 6:**
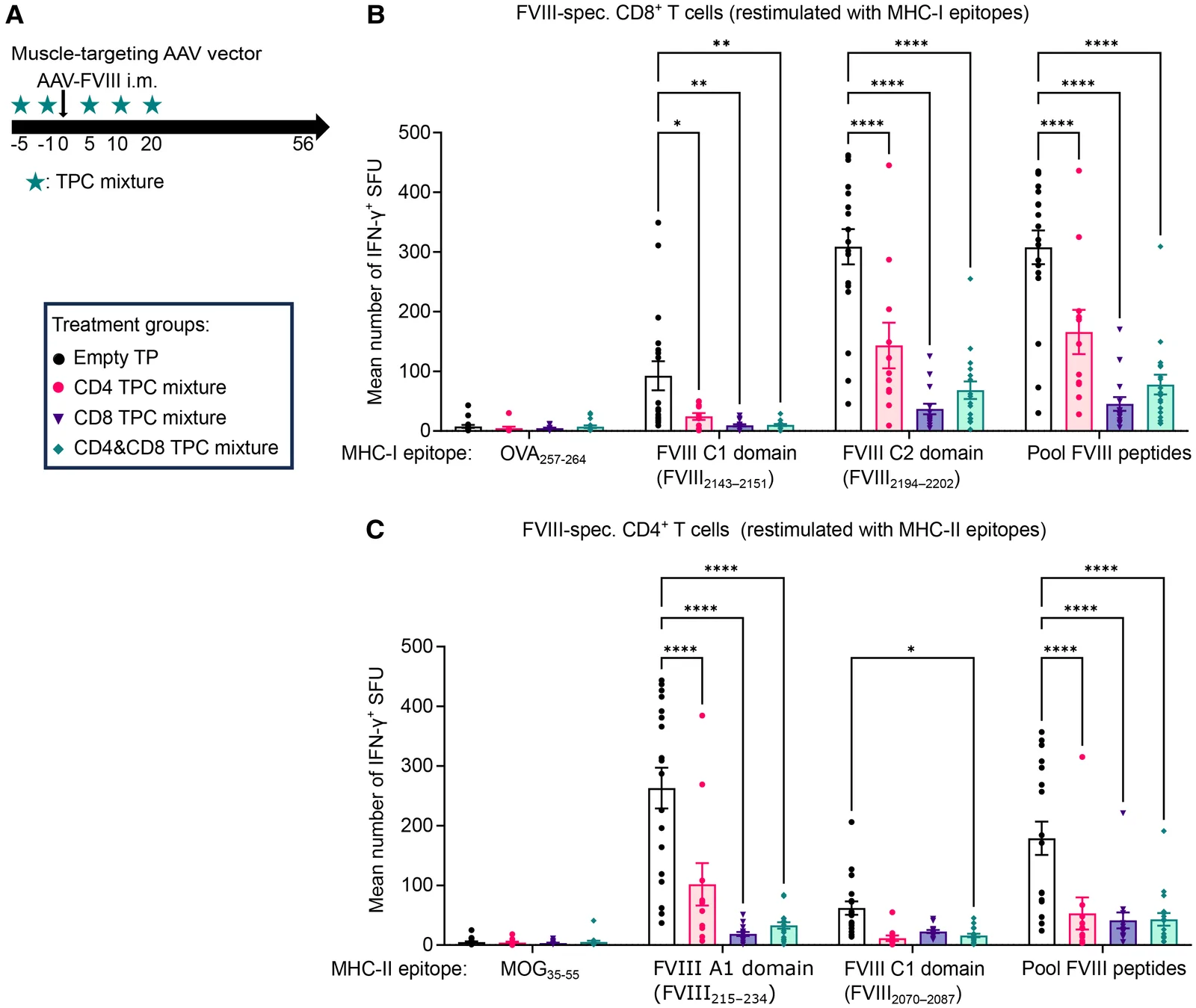
TPCs induce Tspread between CD4^+^ and CD8^+^ T cell subpopulations (A) FVIII knockout mice were i.m. injected at day 0 with an AAV8-FVIII vector (3 × 10^11^ vg/kg) in both gastrocnemii, and each group received the indicated treatment consisting of i.v. injection of empty TPs (black circles), a mixture of TPCs with peptides carrying FVIII-derived CD4^+^ T cell epitopes (CD4 TPC mixture, red circles), FVIII-derived CD8 T cell epitopes (CD8 TPC mixture, purple triangles), or both CD4 and CD8 TPC mixtures (green diamonds) at days −5, −1, 5, 10, and 20 post AAV injection (p.i.). (B and C) Splenocytes were collected at day 56 p.i. and analyzed by *ex vivo* ELISpot assays for their ability to secrete IFN-γ after restimulation with the indicated (B) MHC-I-restricted and (C) MHC-II-restricted peptides. The peptides used for *in vitro* restimulation and their location in the different domains of the FVIII protein are indicated on the *x* axis, and the corresponding specific T cell subsets (i.e., FVIII-specific CD8^+^ or CD4^+^ T cells) expected to be stimulated by these peptides are indicated above each bar graph. As negative controls, the unrelated MHC-I-restricted Ova I peptide (SIINFEKL) and MHC-II-restricted myelin-derived MOG peptide were used. Each group constituted *n* = 11–18 animals from two independent experiments. Graphs show means ± SEM. ∗∗∗∗*p* ≤ 0.0001 by two-way ANOVA with Dunnett’s multiple comparisons test.

To promote T cell tolerization in this setting, we chose to use a TPC mixture containing nanoparticles coupled with either MHC-I- or MHC-II-restricted FVIII-derived peptides, focusing on the most immunogenic CD8^+^ and CD4^+^ T cell epitopes. These immunodominant epitopes were identified beforehand by assessing FVIII-specific CD4^+^ and CD8^+^ T cell responses following immunization of FVIII-deficient mice with AAV8-FVIII followed by *ex vivo* ELISpot assays to evaluate a variety of identified immunogenic FVIII peptides.^46^ Intensity of the T cell responses enabled selection of the immunodominant peptides that were further coupled to TPCs and used for *in vivo* tolerization. Animals were treated with either one of the three selected TPC mixtures: one containing two MHC-I-restricted FVIII peptides, another containing three MHC-II-restricted FVIII peptides, or a combination of both TPC mixtures. In each case, FVIII-deficient mice underwent five i.v. injections of the selected mixture of TPCs at days −5, −1, 5, 10, and 20 and received i.m. injections of AAV8-FVIII at day 0 to provoke an immune response against FVIII (Figure 6A).

The results showed that TPCs induced a significant inhibition of the T cell responses as compared with empty TPs (Figures 6B and 6C). These data were again in line with the TPCs’ efficiency to induce direct tolerization toward the peptide epitopes conjugated to the nanoparticles (i.e., MHC-I-restricted peptides tolerize the CD8^+^ T cells, recognizing the same peptides and MHC-II peptides tolerize the CD4^+^ T cells specific for those peptides). Furthermore, data were also consistent with the induction of Tspread. Indeed, we also observed in this model that the administration of the TPC mixture bearing exclusively specific MHC-I epitopes (i.e., CD8 TPC mixture) significantly reduced CD4^+^ T cell responses (i.e., responses measured *ex vivo* to MHC-II-restricted peptide epitopes) (Figure 6C). Vice versa, administration of the TPC mixture containing exclusively specific MHC-II epitopes (i.e., CD4 TPC mixture) significantly inhibited the responses of CD8^+^ T cells (i.e., responses measured *ex vivo* by ELISpot assays to MHC-I-restricted peptide epitopes) (Figure 6B). These data corroborate the findings described above with Ova and further demonstrate using FVIII that the functional tolerization impact of TPCs *in vivo* extends beyond the conjugated peptide epitopes, spreading to other antigenic epitopes carried by the same viral vector as well as spreading from CD8^+^ T cell subsets to CD4^+^ T cells and vice versa.

### TPC-induced tolerization is antigen specific and does not interfere with vaccination against a tumor antigen

In contrast to general immunosuppression, an advantage of antigen-specific tolerization is its ability to inhibit only unwanted immune responses while maintaining the capacity to respond to vaccination, infection, or tumors. In earlier non-clinical toxicology/safety studies with TPC mixtures carrying peptides related to the autoimmune disease pemphigus vulgaris (TPM201), it was already verified that TPM201 does not impact the immune response to a third-party antigen. In this regard, mice received repeated TPM201 administrations and were then immunized with keyhole limpet hemocyanin (KLH), an immunogenic antigen known to induce a T-cell-dependent antibody response. No TPM201-related influence was reported on the anti-KLH immunoglobulin M (IgM) and IgG levels in male and female animals treated with three different doses of TPM201 (data not shown).

**Figure 7:**
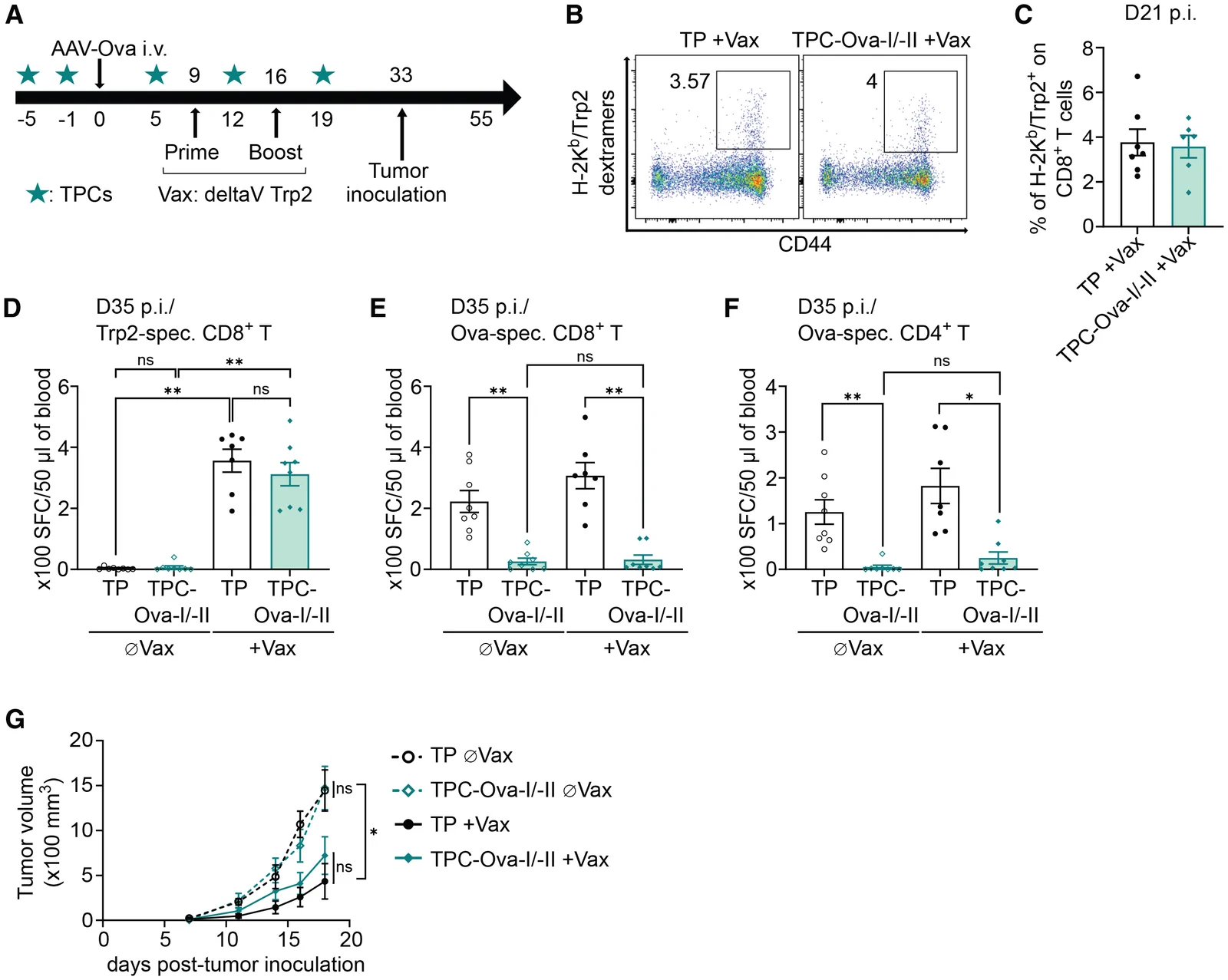
TPC treatment does not impede tumor vaccination or tumor control (A) C57BL/6 mice were i.v. injected at day 0 with AAV8-Ova (5 × 10^11^ vg/kg) and received either empty TPs or TPC-Ova-I/-II i.v. at days −5, −1, 5, 12, and 19 post AAV injection (p.i.). Vaccinated mice (+Vax) were i.m. injected in both gastrocnemii at days 9 and 16 p.i. with 200 μg of deltaV-Trp2 peptide together with 100 μg of poly(I:C); unvaccinated mice (øVax) received PBS. At day 33 p.i., 5 × 10^5^ B16 melanoma cells were s.c. inoculated. (B) Representative flow-cytometric panel of H-2K^b^/Trp2_180–188_ dextramer staining at day 21, and (C) frequency of Trp2-specific CD8^+^ T cells at day 21. (D–F) At day 35, ELISpot assays were performed from blood samples, and circulating T cells were evaluated for their ability to secrete IFN-γ after restimulation with (D) MHC-I-restricted Trp2_180–188_, (E) MHC-I-restricted Ova-I, and (F) MHC-II-restricted Ova-II peptides. The corresponding specific T cell subsets expected to be restimulated by these peptides are indicated above each of these bar graphs (D–F), and the treatment received by each group is indicated on the *x* axis. (G) Tumor volumes were followed over time in each indicated group of mice inoculated with B16 melanoma cells at day 33. The treatment received in each group consisted of empty TPs without vaccination (open circles), TPC-Ova-I/-II without vaccination (open cyan diamonds), TP with vaccination (black circles), and TPC-Ova-I/-II with vaccination (cyan diamonds). Each group constituted *n* = 8 animals. Graphs show means ± SEM. ns, not significant; ∗*p* ≤ 0.05, ∗∗*p* ≤ 0.01, ∗∗∗*p* ≤ 0.001 by two-way ANOVA.

To further demonstrate that tolerization with TPCs does not result in general immunosuppression in the context of AAV-mediated GT, we investigated how TPC-mediated tolerization toward the epitopes carried by the AAV vector affects the immune response to an unrelated antigen (i.e., not carried or expressed by the viral vector). To this end, we applied TPC-Ova-I/Ova-II in mice injected i.v. with AAV8-Ova and additionally subjected the mice to an anti-tumor peptide vaccination against the Trp2 antigen found in melanoma^47^ (Figure 7A). Even if peptide vaccination against the Trp2 antigen was initiated during the tolerization phase toward Ova antigens, the data confirmed that TPC-Ova-I/Ova-II injections did not interfere with the CD8^+^ response to Trp2 (Figures 7B and 7C). Evaluation of the immune responses by ELISpot further confirmed that the TPC-Ova-I/Ova-II tolerized group exhibited a level of anti-Trp2 CD8^+^ reactive cells comparable to that in the non-tolerized control group (Figure 7D). Moreover, TPC-Ova-I/Ova-II-mediated tolerization led to a potent blockade of CD8^+^ and CD4^+^ T cell responses against Ova epitopes in both vaccinated and unvaccinated groups (Figures 7E and 7F). Next, we monitored the partial control of tumor growth conferred by Trp2-peptide vaccination^47^ by inoculating the aggressive B16 melanoma cell line 2 weeks after the Trp2-peptide boost (Figure 7A). TPC-Ova-I/Ova-II-tolerized and non-tolerized Trp2-vaccinated mice exhibited comparable levels of partial control of tumor growth relative to unvaccinated mice (Figure 7G). These data illustrate the antigen specificity of the TPC-mediated tolerization, which effectively inhibits immune responses against the antigens carried by the vector without affecting the immune system’s capability to respond to vaccination.

## Discussion

Antigen-specific tolerization against unwanted immune responses is needed for safe, predictable, and durable outcomes in a broad spectrum of clinical situations, including AAV-mediated GT.^48^ Addressing this medical need, we developed a nanoparticle-based platform to selectively deliver disease-relevant T cell epitopes to LSECs and exploit their unique potential for inducing antigen-specific tolerance. This novel strategy has demonstrated proof of concept in a variety of preclinical studies^8^^,^^9^^,^^10^ and in a clinical study of patients with celiac disease.^49^ Indeed, TPCs coupled to gluten-derived peptides demonstrated safety and significant dose-dependent functional suppression of gluten-specific T cells.^49^ The aim of the present study was to further assess whether TPCs also have the capacity to tolerize unwanted T cell responses in the context of viral-vector GT and to precisely evaluate the relationship between the peptide epitopes used for tolerization and the final outcome (tolerization to one or multiple other antigens derived from the capsid or the transgene). To this end, we used different immunogenic models of muscle- or liver-directed GT based on different serotypes of AAV vectors coding either for Ova or for FVIII. We selected highly immunogenic antigen models (including the xenogeneic secreted Ova protein) and immunogenic anatomical sites of injection (i.e., muscle site is more immunogenic than the liver) to evaluate the tolerization potential of our approach under stringent conditions. Our data demonstrate that TPC administration not only significantly induces antigen-specific tolerance toward the peptides coupled to the nanoparticles but also promotes remarkable Tspread, resulting in a broader tolerance of CD4^+^ and CD8^+^ T cell responses to other relevant capsid- and transgene-derived antigens as well as improved transgene expression over time.

The TPC-based platform is designed to deliver antigen-derived peptide epitopes primarily to LSECs, as evidenced by our biodistribution data based on TPCs coupled to a fluorescent peptide tracer. Although TPCs may also be marginally captured by Kupffer cells, which are known to capture circulating nanoparticles, our previous and present data indicate that TPCs are predominantly taken up by LSECs. In previous studies, we have shown that, following their uptake, the TPCs are processed and their carried peptides readily released inside LSECs within 1 h^8^ to be presented by the appropriate MHC molecules at the LSEC surface.^9^ We believe that targeting LSECs, rather than other NPLCs, represents a potentially superior strategy.^50^^,^^51^ In fact, LSECs may appear even superior to classical APCs, as they have been reported to exhibit 10-fold higher antigen-uptake capabilities and 2-fold higher cross-presentation efficiency in comparison to dendritic cells.^2^ Additionally, LSECs are at the crossroads of multiple tolerization mechanisms, promoting Treg expansion, production of immunoregulatory interleukin-10 (IL-10) and transforming growth factor-β (TGF-β) cytokines, PD1/PD-L1 signaling, T cell silencing, and sequestration or deletion of activated CD8^+^ T cells.^2^^,^^3^ Also, as LSECs maintain their tolerogenic function even under moderate inflammatory conditions, they represent a potentially more robust and safer cell target for tolerance induction compared to other tolerogenic cells.^1^^,^^5^^,^^6^^,^^7^

To assess the tolerization efficacy of TPCs *in vivo*, we used AAV vectors coding for Ova or FVIII, two proteins known to elicit significant immune responses. Indeed, AAV vectors coding for these proteins are known to induce robust T-cell-dependent immune responses in animal models, leading to limited transgene persistence.^30^^,^^31^^,^^42^^,^^43^^,^^44^^,^^45^ Our results demonstrate significant inhibition of T cell immune responses in both models (i.e., using AAV coding for Ova or FVIII) and robust TPC-mediated tolerization in different settings (i.e., liver or muscle GT).

Using the AAV-Ova model where the immune responses can readily be monitored, our data demonstrate that TPCs coupled with peptides derived from the transgene product Ova and/or from the AAV capsid significantly inhibit cellular immune responses targeted by the TPCs and notably suppress the emergence of specific and functional CD8^+^ T cells. This was evidenced by a significant reduction in transgene-specific cytotoxic CD8^+^ T cells in blood, liver, and spleen following muscle and liver GT, accompanied by phenotypic modulation of the few remaining specific CD8^+^ T cells; and a reduction in their functional capacity to secrete IFN-γ upon *in vitro* restimulation, consistent with anergy or exhaustion. This is in line with data showing that CD8^+^ T cells that were initially activated by cross-presenting LSECs rapidly return to a quiescent state and become refractory to further TCR stimulation.^4^ Importantly, these TPC-mediated effects support sustained transgene expression, highlighting the impact of TPC-mediated tolerization on GT.

**Figure 8:**
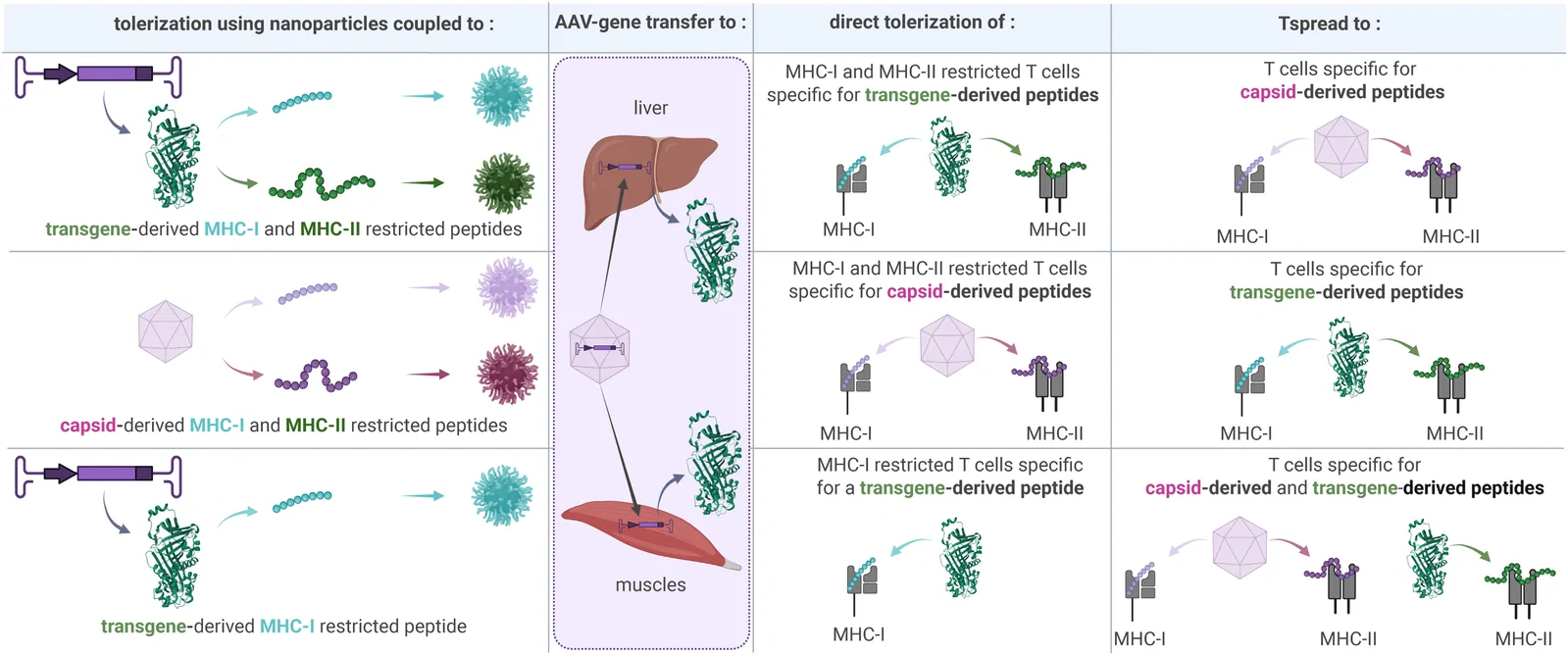
Depiction of the different modalities by which Tspread can extend tolerization to other antigens and other T cells Schematic depiction of different nanoparticle-mediated tolerization modalities leading to tolerance spreading (Tspread) as observed in the present study using AAV-mediated gene transfer of Ova transgene. Nanoparticles coupled with selected peptide epitopes were designed and injected *in vivo* using the i.v. route to preferentially target tolerogenic liver sinusoidal endothelial cells (LSECs). Evaluation of this tolerization strategy *in vivo* in the context of muscle or liver AAV-mediated gene therapy showed efficient direct T cell tolerization against the selected epitopes (i.e., epitopes conjugated to the nanoparticles) but, remarkably, also toward other epitopes through tolerance spreading (Tspread). At least three situations of Tspread have been observed during the present study using the AAV-Ova model and are illustrated here: (1) direct and targeted T cell tolerization toward MHC-I- and MHC-II-restricted epitopes derived from the transgene resulted in Tspread to additional MHC-I- and MHC-II-restricted capsid-derived epitopes; (2) vice versa, direct and targeted T cell tolerization toward MHC-I- and MHC-II-restricted epitopes derived from the capsid resulted in Tspread to additional MHC-I- and MHC-II-restricted transgene-derived epitopes; and (3) direct and targeted T cell tolerization toward a single MHC-I-restricted epitope derived from the transgene resulted in Tspread to additional MHC-II-restricted transgene-derived epitopes and to MHC-I- and MHC-II-restricted capsid-derived epitopes. Hence, Tspread can extend tolerance to a broader range of antigens and T cell subsets, encompassing both CD4^+^ (MHC-II-restricted) and CD8^+^ (MHC-I-restricted) T cell subsets, offering the possibility to strengthen tolerance to both capsid- and transgene-derived antigens. Additionally, in the context of AAV vector coding for FVIII, we could further confirm and demonstrate that direct and targeted T cell tolerization toward exclusive MHC-I-restricted epitopes derived from the FVIII resulted in Tspread to MHC-II-restricted FVIII-derived epitopes, and vice versa (not depicted here for simplicity). Created in BioRender. Adriouch, S. (2025) https://BioRender.com/a40h609.

As TPCs are coupled to defined peptide epitopes known to bind either MHC-I or MHC-II molecules, the tolerizing effects toward the targeted and non-targeted epitopes can be distinctively investigated using *ex vivo* restimulation assays with peptides derived from the same or from different antigens. Remarkably, in addition to the direct tolerization of the T cells specific for the targeted epitopes (i.e., same peptide epitopes as those coupled to the TPCs), we also observed concomitant tolerization toward non-targeted epitopes (i.e., tolerance to other epitopes different from those coupled to the TPCs), as confirmed here in two different experimental settings (i.e., using AAV vectors coding for either Ova or FVIII). Interestingly, spreading of tolerance to these other non-targeted epitopes appears to be related to both “linked” or “bystander” suppression,^52^ defined as a situation where tolerance toward one given antigen is transferred to another linked/bystander antigen co-expressed by the same cell and/or within the same microenvironment, as well as to “infectious tolerance” or “cross-tolerization” effects,^53^ defined as the active spreading of tolerance from one given T cell subset to another. However, these concepts remain ill defined at the molecular level and conceivably involve common cellular and molecular mechanisms, including CD4^+^ and CD8^+^ Tregs, expression of immunoregulatory receptors and cytokines, downmodulation of co-stimulatory signaling molecules, and possibly local metabolic modifications.^52^^,^^53^^,^^54^^,^^55^^,^^56^^,^^57^ As this extension of tolerance described herein involves the dissemination of immune tolerance both to other antigens and to other T cell subsets, we proposed to refer to this mechanism as tolerance spreading and coined the term Tspread. Dissemination of tolerance to other antigens (known as linked/bystander suppression) has been already reported, for instance in allograft transplantation, and extension of tolerance to other T cell subsets through “infectious tolerance” has been described in peptide immunotherapy for allergic diseases.^52^^,^^53^^,^^54^^,^^55^^,^^56^^,^^57^ To the best of our knowledge, the combination of both—here defined as Tspread—has not been reported so far. Importantly, our data demonstrate the induction of Tspread following nanoparticle-mediated LSEC targeting not only in the context of liver GT but also in the more immunogenic situation of i.m. AAV injection. Indeed, nanoparticles coupled to transgene-derived epitopes induced Tspread toward the non-targeted AAV capsid antigens and vice versa (Figures 5B, 5C, and 8). Most importantly, it is reported here for the first time that targeted tolerization toward an exclusive MHC-I-restricted transgene-derived epitope extends beyond this single epitope to broadly tolerize MHC-I- and MHC-II-restricted epitopes derived from both the transgene and the vector capsid (Figures 5E–5F and 8). Although not formally excluded, it appeared rather unlikely here that Tspread relies on direct transduction of LSECs by AAV vectors, as we observed this mechanism of Tspread also when we used i.m. injection of AAV8 or AAV1 vectors. Tspread likely relies on the presentation of different antigens by the same APCs. Our data suggest that TPCs promote LSEC-mediated activation of Foxp3^+^ Tregs and expansion of CD8^+^LAP^+^ Tregs, which are conceivably involved in broadening tolerization *in vivo*.^58^^,^^59^ Extending tolerance through Tspread to other epitopes and antigens and to other T cell subsets is of utmost relevance *in vivo* to achieve a robust and durable state of tolerance. This finding is of clinical importance, particularly in contexts where tolerization to both the capsid and the transgene corresponds to unmet medical needs.^14^^,^^22^^,^^60^^,^^61^^,^^62^

We provide evidence that the mechanisms of action underlying the TPC-induced tolerization include (1) activation of Foxp3^+^CD4^+^ Tregs, as suggested by the statistically significant increase in Ki-67^+^ Tregs; (2) inhibition of the clonal expansion and/or deletion of transgene-specific CD8^+^ T cells in blood/liver/spleen; (3) phenotypic and functional exhaustion of transgene and capsid-specific T cells, and (4) upregulation of immunoregulatory markers on CD8^+^ transgene-specific T cells. TPC treatment induced upregulation of CD73 on antigen-specific CD8^+^ T cells, an ectoenzyme involved in the generation of extracellular adenosine, a well-known potent immunosuppressive molecule.^40^^,^^41^ Also, our data suggested that TPC administration is associated with the upregulation (though not reaching statistical significance) of LAP on the surface of CD8^+^ T cells, corresponding to the processed C-terminal portion of TGF-β precursor. Apart from its critical role in differentiation of CD4^+^ Tregs, TGF-β is also known to suppress CD8^+^ T cell activation and function through direct and indirect pathways.^63^^,^^64^ Interestingly, LAP^+^ lymphocytes displaying membrane-bound TGF-β (including CD4^+^, CD8^+^, and γδ T cells) correspond to Tregs that do not necessarily express Foxp3.^63^^,^^65^^,^^66^^,^^67^ Accordingly, CD8^+^LAP^+^ T cells have been described as regulatory/suppressive cells in the context of autoimmune diseases, transplantation, or cancer.^36^^,^^68^^,^^69^ Akin to CD4^+^Foxp3^+^ Tregs, CD8^+^LAP^+^ T cells represent an important subset of Tregs involved in long-term tolerance. As mentioned before, LSECs represent the major NPLCs responsible for the induction of hepatic Tregs.^70^ However, LSEC-induced Tregs may not necessarily express Foxp3, which may cause an underestimation of their number in the liver.^71^ Our data suggest that LSECs induce not only CD4^+^Foxp3^+^ Tregs but also CD8^+^ LAP^+^ Tregs, most likely in a TGF-β-dependent manner.

As opposed to general immunosuppression, one key objective of tolerization protocols is to achieve antigen-specific inhibition of immune responses without compromising the immune response to vaccine and the anti-tumoral immune response. We demonstrate tolerization toward capsid- and transgene-derived antigens without impairing vaccination against a tumor antigen. This indicates that TPC-mediated tolerization is not associated with general immunosuppression but genuinely relies on antigen-specific mechanisms. In contrast to nanoparticles loaded with immunosuppressive drugs,^72^ the LSEC-targeted antigen-specific strategy might reduce the risk of tolerizing against any given vaccine- or tumor-derived antigen. Moreover, we demonstrate that TPC-based tolerization and vaccination can be applied concomitantly, without any interference with tolerization efficiency or immunization efficacy against the vaccine antigen. This is likely not the case when using nanoparticles loaded with immunosuppressive drugs, which are taken up more broadly by different types of APCs, whereby the vaccination needs to be applied successively rather than concomitantly.^72^ Importantly, applying TPC-mediated antigen-specific tolerance induction and vaccination at the very same time, represents a better model to evaluate the specificity and safety of our approach and to reflect the real-world scenario, where the immune system may be confronted to multiple antigens simultaneously. However, beyond demonstrating that the anti-tumor response is not affected, it remains to be established in future studies that TPC-mediated antigen-specific tolerization likewise does not interfere with the immune responses against pathogens. In addition, species differences may limit extrapolation from non-clinical models, underscoring the need for human data. As first evidence, we reported that upon treatment of celiac patients with a TPC mixture containing gluten-derived peptides, antigen-specific tolerance was confirmed in patients, without evidence of increased susceptibility to infections or signs of generalized immunosuppression noted during these early studies (TCeD21 study, EudraCT Number 2022-001656-41, ClinicalTrials.gov ID NCT05660109).^73^^,^^74^

Other comprehensive sets of experiments on TPC-mediated suppression of antibody responses lie beyond the scope of the present study, which was limited to TPC-mediated tolerization of cellular T responses and to the first demonstration of a Tspread effect across antigen types (transgene-derived to capsid) and between T cell subsets (CD8^+^ and CD4^+^ T cells). Notwithstanding, controlling the humoral responses against the AAV vector and against the transgenic protein represents another important aim for clinical translation. However, such comprehensive future study should evaluate the influence of multiple factors that may impact the efficacy of tolerization, such as the antigen immunogenicity and location (secreted, membrane, or cytosolic), the antigen dose and persistence, the AAV injection site, MHC affinity of the peptide epitopes carried by the TPCs, and precise and longitudinal evaluation of the number and function of antigen-specific T follicular helper cells (T_FH_) to precisely evaluate T_FH_-B cell cooperation. Yet, results from other preclinical studies and the clinical study in pemphigus vulgaris demonstrate that TPCs, coupled to peptide epitopes targeting CD4^+^ T cells, indeed have a suppressive effect on disease-relevant humoral responses, most probably by inhibiting T cell-B cell cooperation.^75^

In conclusion, nanoparticle-mediated peptide delivery targeted to LSECs, in the context of AAV GT, induced robust tolerization to defined T cell epitopes along with remarkable Tspread to a broader range of antigens conveyed by the same viral vector and involved tolerization of both CD4^+^ and CD8^+^ T cell subsets. This novel approach used either alone or in combination with other approved treatment might therefore open new avenues to promote tolerance to both capsid- and transgene-derived epitopes, improve transgene persistence, and enhance safety in the context of AAV-mediated GT to humans.

## Materials and methods

### Animals used in the study

Female C57BL/6Rj (B6) mice were obtained from Janvier Labs at 6–7 weeks of age and were used between 8 and 12 weeks of age at the beginning of each experiment. TCR-transgenic OT-I mice and OT-II mice, along with congenic B6 mice expressing the CD45.1 (Ly5.1) allelic marker, were obtained from Charles River Laboratories. These mice were crossed to generate [OT-I × CD45.1] and [OT-II × CD45.1] mouse strains, which were used as a source of anti-Ova TCR-transgenic OT-I CD45.1^+^CD8^+^ T cells or OT-II CD45.1^+^CD4^+^ T cells, respectively. Mice were housed in a specific pathogen-free barrier facility. Of note, for all studies involving AAV vectors coding for Ova, previous studies using male and female did not show any difference in the immune responses induced by their i.m. injections.^31^^,^^76^ We therefore used in our present study only female mice when AAV-Ova vectors were injected i.m. to reduce the number of animals and the variability related to the aggressive male behavior, sometimes resulting in skin lesions and premature removal from the study. On the contrary, in all experiments involving AAV8 vectors injected i.v., to target the liver, only male mice were used, as the transduction efficiency of the liver has been reported to be around ten times more efficient in males than in females.^77^

Male and female HemA (*F8*-knockout resulting in FVIII-deficiency) mice (genetic background C57BL/B6;129S-F8tm1Kaz/J) of 6–8 weeks of age were obtained from CDTA-Orléans (and housed and use in the accredited animal facility of CEF-UMRS 1138). Mice received i.m. injections of AAV8 vectors carrying FVIII transgene (3 × 10^11^ viral genomes per mouse [vg/mouse]) at day 0, while control animals received injections of PBS. The number of animals per group ranged from four to ten and is specified in the corresponding figure legends. All experimental procedures were conducted with operators blinded to the treatment groups.

All animal experiments were approved by the ethical committee and the French Ministry of Education and Research (APAFIS #19709, APAFIS #35903) as well as the respective review boards of University Medical Center Hamburg-Eppendorf, Germany (no. N032/2018). All mice were bred and housed in an accredited animal facility (C765407 or C-75-06-12) under specific pathogen-free conditions. All the animal procedures adhered strictly to both local and national guidelines in agreement with the European regulations, and all possible efforts were taken to minimize any animal suffering.

### Production of recombinant AAV vectors for muscle or liver transduction

The initial plasmid construct used for the generation of the transgene coding for Ova has been described previously.^30^^,^^31^ In brief, this plasmid encodes a secreted form of Ova under the transcriptional control of the ubiquitous cytomegalovirus (CMV) promoter. To produce AAV8-Ova, the construct was recloned into a pFB plasmid before production, purification, and titration, all performed by Virovek (Houston, USA) using the baculovirus expression system in Sf9 insect cells as described previously.^78^ We injected the AAV8 vectors either i.m., to target skeletal muscle cells, or i.v., to target mainly hepatocytes. For muscle transduction, mouse hind legs were shaved under light anesthesia, and AAV8-Ova was injected into gastrocnemius muscle sites (25 μL per injection site) to reach the indicated dose specified in vg/mouse). For liver transduction, mice were i.v. injected with AAV8-Ova vector diluted in 100 μL of PBS following light anesthesia. For the FVIII-specific proof-of-concept studies presented here, the same transgene as described previously, termed codop-hFVIII-V3, was used.^79^ The corresponding AAV8-CMV-codop-hFVIII-V3 vector was produced by Virovek (Houston, USA) as described above. This vector was administered i.m., as described above, at a dose of 3 × 10^11^ vg/mice.

### TP and TPC preparation for TPC-mediated tolerization

TPs were produced by coating oleic acid-stabilized superparamagnetic iron oxide nanoparticles (SPIONs) with an amphiphilic polymer (poly(maleic acid-alt-1-octadecene)), as described by Carambia et al.^9^ TPs were conjugated with synthetic Ova-derived peptides and AAV-capsid-derived peptides in the presence of 1-ethyl-3-(3-dimethylaminopropyl)-carbodiimide (EDC), with 150-fold excess of the respective peptide, and incubated for 2.5 h to form TP-loaded tolerizing particle conjugates (TPCs). Free peptide and EDC were removed under centrifugal force using a spin filter (100 kDa, 4,200 rpm, 25°C). Each TPC contained up to 100 molecules of a conjugated peptide. Peptide content and coupling efficiency were determined using the AccQ Tag derivatization method for amino acid analysis (Waters). For peptides with unfavorable conjugation characteristics identified via *in silico* analysis, a proprietary linker technology was applied to enhance coupling efficiency. TPCs were diluted in 5 mM Tris-mannitol-lactate formulation buffer and used at a dose of 14 nmol per peptide and injection. To induce antigen-specific tolerization, TPCs coated with the indicated peptides, restricted by either MHC-I as Ova-I (SIINFEKL), PL8 (PQYGYLTL), and ML8 (MIPQYGYL), or by MHC-II as Ova-II (ISQAVHAAHAEINEAGR), and RK15 (RNSLANPGIAMATHK), were mixed and i.v. injected in a volume of 150 μL of formulation buffer. Unless otherwise stated, injections of TPCs were repeated five times, between days −5 or −1 to around day 20 post AAV administration, as indicated in the figures for each experiment. While two injections at day −7 and day 0 were sufficient to significantly induce tolerance, increasing the frequency of administration to five injections (with the last injection on days 19–21) allowed the persistence of the tolerization effect for up to 103 days post treatment in our studies. As negative controls, we previously tested vehicle (formulation buffer), peptide-free nanoparticles (TPs), or TPCs conjugated with irrelevant (mock) peptides. Our results indicated that these negative controls did not significantly differ from each other. For practical reasons, we therefore used TPs in the present studies.

For the studies involving TPCs coupled with FVIII peptides, relevant CD4^+^ and CD8^+^ T cell epitopes were preselected based on published studies that have identified the immunodominant peptides recognized in patients or in HLA-humanized mice (notably according to Steinitz et al.^46^ and Pratt and Thompson^80^). From this preselected set of 14 immunodominant peptide epitopes derived from the human FVIII protein, located in different regions of the B-domain-deleted therapeutic protein (BDD-FVIII) and predicted to be also presented by the mouse MHC molecules, each peptide epitope was directly evaluated in T cell restimulation assays (ELISpot assays after *ex vivo* peptide restimulation) for the capacity to stimulate IFN-γ secretion using T cells collected from FVIII-deficient mice immunized with AAV8-FVIII vector. The resulting most immunodominant peptides include three CD4^+^ T cell epitopes each modified by a short N-terminal linker to improve TPC coupling: (LPVDARFPPRVPKSFPFNTS derived from the A1 domain of human FVIII, IKHNIFNPPIIARYIRLH derived from the C1 domain of human FVIII, and TASSYFTNMFATWSPSKARLUM derived from the C2 domain of human FVIII) as well as two CD8^+^ T cell epitopes each modified by a short N-terminal linker to improve TPC coupling (THYSIRSTL derived from the C1 domain of human FVIII and SMYVKEFLI derived from the C2 domain of human FVIII). As negative controls in the ELISpot assays, unrelated peptides were used for *ex vivo* stimulation, i.e., Ova-I peptide (SIINFEKL) as an MHC-I-restricted peptide and myelin oligodendrocyte glycoprotein (MOG) peptide (MEVGWYRSPFSRVVHLYRNGK) as an MHC-II-restricted peptide.

### Intravital microscopy

Intravital microscopy was carried out as previously described.^81^ In brief, C57BL/6 mice underwent inhalation anesthesia with 2% isoflurane (at 2 L/min) via a small animal anesthetic face mask. The tail vein was catheterized, skin and peritoneum were opened, and the liver was prepared and attached to a coverglass. Imaging was performed using a Nikon A1 confocal microscope with a resonant scanner for image acquisition at 30 frames per second. To visualize TPC targeting, Cy5-labeled nanoparticles were injected via the inserted tail vein catheter followed by monitoring of their uptake into the liver for up to 15 min.

### Flow-cytometry analysis of cell subsets within the liver targeted by fluorescently labeled TPCs

For evaluation of the major cell types targeted by TPCs within the liver, NPLCs were isolated by density gradient centrifugation 10 min after i.v. administration of the fluorescently labeled TPC-Ova-II-Cy5 nanoparticles. In brief, mouse livers were perfused with 1 mg/mL collagenase type 2 (Worthington) in Gey’s balanced salt solution, mechanically dissected, and digested for 25 min at 37°C in 1 mg/mL collagenase type 2 solution. Hepatocytes were removed by centrifugation, and NPLCs were recovered using a 17% Optiprep (Sigma-Aldrich) gradient centrifugation. Cells were then stained and analyzed by flow cytometry.

### Adoptive transfer of anti-Ova TCR-transgenic T cells

TCR-transgenic Ova-specific CD8^+^ T cells and CD4^+^ T cells were obtained, respectively, from the spleens of [OT-I × CD45.1] and [OT-II × CD45.1] mice and purified by magnetic sorting using negative isolation kits (Invitrogen, Thermo Fisher). More than 95% of the purified cells displayed the expected CD3^+^CD19^−^Vα2^+^Vβ5^+^CD45.1^+^ phenotype, in addition to the expression of CD4 or CD8, as assessed by flow cytometry. Cells were washed and mixed at a 1:1 ratio, and the total 2 × 10^5^ cells were then adoptively transferred i.v. into each B6 recipient mouse.

### Antibodies and flow cytometry

Fluorochrome-conjugated antibodies were all obtained from Sony Biotechnology, and cell staining was performed using standard methods. In brief, all incubations with antibodies were carried out at 4°C for 20 min, followed by washing steps and cell resuspension with FACS buffer (3% BSA and 1 mM EDTA in PBS). Intracellular Foxp3 and, Ki-67 staining were performed using the intracellular fixation and permeabilization kit from eBioscience and Thermo Fisher Scientific.^82^^,^^83^ PE-conjugated H-2K^b^/Ova-I or H-2K^b^/Trp2_180–188_ dextramers were used to detect CD8^+^ T cells that specifically recognize the immunodominant Ova_257–264_ (Ova-I) or Trp2_180–188_ peptides using the manufacturer’s protocol (Immudex). Similarly, single-cell suspensions derived from spleen, liver, or peripheral blood (PBL) were analyzed by flow cytometry using an LSRFortessa cytometer (BD Biosciences) using FlowJo software (Tree Star).

### Peptide vaccination and tumor model

Peptide vaccination was performed by injection of 200 μg of deltaV-modified Trp2_180–188_ peptide (delta-V Trp2, SIYDFFVWL), found to generate a stronger immune response^47^ than the native Trp2_180–188_ peptide (SVYDFFVWL). The deltaV-Trp2 peptide was mixed with 100 μg of poly(I:C) before i.m. injection. Injection was repeated 7 days after the first immunization to boost the immune response. For tumor inoculation, mice were injected subcutaneously (s.c.) with 10^5^ B16 melanoma cells in their shaved flanks. Mice were then examined every other day, and tumor development was monitored using a digital caliper as described previously.^82^^,^^83^ Tumor volume was estimated using the following formula: 0.5 × length × width^2^.

### ELISA and ELISpot assays

Titration of anti-Ova IgG antibodies was performed by ELISA as previously described.^31^^,^^76^ Anti-Ova IgG titers were defined as the dilution yielding the half-maximum optical density obtained with a positive control serum that was used throughout the entire study. ELISpot assays were used to quantify the numbers of Ova-specific CD8^+^ or CD4^+^ T cells secreting IFN-γ upon *in vitro* peptide restimulation as previously described.^31^^,^^76^ For that, 10^5^ to 2.5 × 10^5^ splenocytes per well were cultured overnight in RPMI medium in the presence of 10 μg/mL Ova-I (SIINFEKL) or Ova-II (ISQAVHAAHAEINEAGR) peptides for detection of, respectively, MHC-I- or MHC-II-restricted CD8^+^ and CD4^+^ immune responses directed against Ova, or with PL8 (PQYGYLTL) peptide for detection of MHC-I-restricted CD8^+^ AAV-specific immune responses, or with RK15 (RNSLANPGIAMATHK) peptide for detection of MHC-II-restricted CD4^+^ AAV-specific immune responses.^31^^,^^76^ For the experiment involving Trp2-melanoma antigen vaccination,^47^ restimulation was performed using the MHC-I-restricted Trp2_180–188_ peptide (SVYDFFVWL). The numbers of spots in each well were analyzed with an ELISpot plate reader and the ImmunoSpots software (C.T.L.).

For the experiments with FVIII-directed tolerization, ELISpot assays were used to quantify the numbers of FVIII-specific CD8^+^ or CD4^+^ T cells secreting IFN-γ upon *in vitro* restimulation. To this end, the plates were coated with capture anti-mouse IFN-γ antibodies, and 6 × 10^5^ splenocytes per well were cultured for 24 h or 96 h in RPMI medium (supplemented with mouse IL-2 at 50 IU/mL for 96 h of stimulation) in the presence of 10 μg/mL FVIII-derived or control peptides for detection of MHC-I- or MHC-II-restricted cellular immune responses. Full-length FVIII and concanavalin A were used as positive controls. Spots were detected with a biotin-conjugated anti-mouse IFN-γ followed by streptavidin-peroxidase. The spots were revealed with TMB substrate according to the manufacturer’s instructions (Mabtech, Sweden). Numbers of spots in each well were analyzed with an ELISpot plate reader and dedicated ImmunoSpots software using the SmartCount and Autogate functions (CTL, Shaker Heights, OH, USA).

### Quantification of Ova mRNA in transduced muscles and transduced livers

Ova DNA and corresponding Ova mRNA from transduced muscles and livers were quantified by real-time PCR using SYBR Green Mastermix (Roche) in a final volume of 10 μL as described previously.^31^ All qPCRs were performed with a LightCycler 480 (Roche). The relative amount of Ova mRNA was determined using a standard curve obtained after serial dilutions of a plasmidic construct coding for Ova and normalized for each sample by the amount of Eef2 and GAPDH.

### Statistical analysis

All data are shown as mean values, and error bars represent SEM. The non-parametric Mann-Whitney test was used to compare differences between two experimental groups. For multigroup comparison, one-way ANOVA followed by Dunnett’s post hoc test was used. For kinetics, two-way ANOVA with Dunnett’s multiple comparisons test was performed. Unless otherwise stated, all the experiments have been repeated at least three times, and illustrations shown here are representative of all experiments. Differences were considered statistically significant when *p* values were less than 0.05 (∗), 0.01 (∗∗), or 0.001 (∗∗∗). All calculations were performed using Prism software (GraphPad).

## Data and code availability

The data that support the findings of this study are available from the corresponding authors upon reasonable request.

## Acknowledgments

We would like to thank the flow-cytometry facility of the 10.13039/100009942Institute for Research and Innovation in Biomedicine (IRIB) for technical expertise, and University Rouen Normandie, 10.13039/501100001677INSERM, and TOPAS Therapeutics for starting funds and infrastructures. We thank the staff from the Cordeliers functional Experimentation (CFE) at 10.13039/100018050Centre de Recherche des Cordeliers (Paris) for assistance.

## Author contributions

S.A. and S.F. led and co-supervised the project; R.H., S.-H.W., S.D., M.B., M.H., and C.G. performed the experiments; R.D., L.M.M.M., M.F., A.-L.V., and D.M. designed and generated the nanoparticle-peptide conjugates; R.H., S.D., S.L.-D., S.A., and S.F. designed the experiments; R.H., M.B., S.-H.W., S.D., S.F., and S.A. plotted and analyzed the data; R.H., S.-H.W., S.F., and S.A. drafted the manuscript; and all co-authors contributed to interpretation of the data and revision and finalization of the manuscript.

## Declaration of interests

S.A., S.F., R.H., and R.D. are co-inventors of a pending patent (EP 24 306 311.2 / WO2026027739A1) covering the use of TPC- mediated tolerance induction in the context of AAV GT.

## Supplemental information

Document S1. Figure S1

Document S2. Article plus supplemental information
