# Supplementary material for: Nanoparticles targeting liver sinusoidal endothelial cells improve tolerance to vector and transgene antigens through tolerance spreading
Source: Mol Ther. 2026 Mar 12;34(7):3854–71. doi: 10.1016/j.ymthe.2026.03.009 (PMC13330011; doi:10.1016/j.ymthe.2026.03.009)
Supplement: Supplementary file 1 [file mmc1.pdf]

YMTHE, Volume *34*

Supplemental Information

Nanoparticles targeting liver sinusoidal endothelial cells improve tolerance to vector and transgene antigens through tolerance spreading

Romain Hardet, Shu-Hung Wang, Sandrine Delignat, Marine Blandin, Reinaldo Digigow, Cornelia Gottwick, Markus Heine, Lígia Margarida Marques Mesquita, Marco Fanzutti, Anna-Lisa Vocaturo, Disha Mungalpara, Olivier Boyer, Sébastien Lacroix-Desmazes, Sabine Fleischer, and Sahil Adriouch

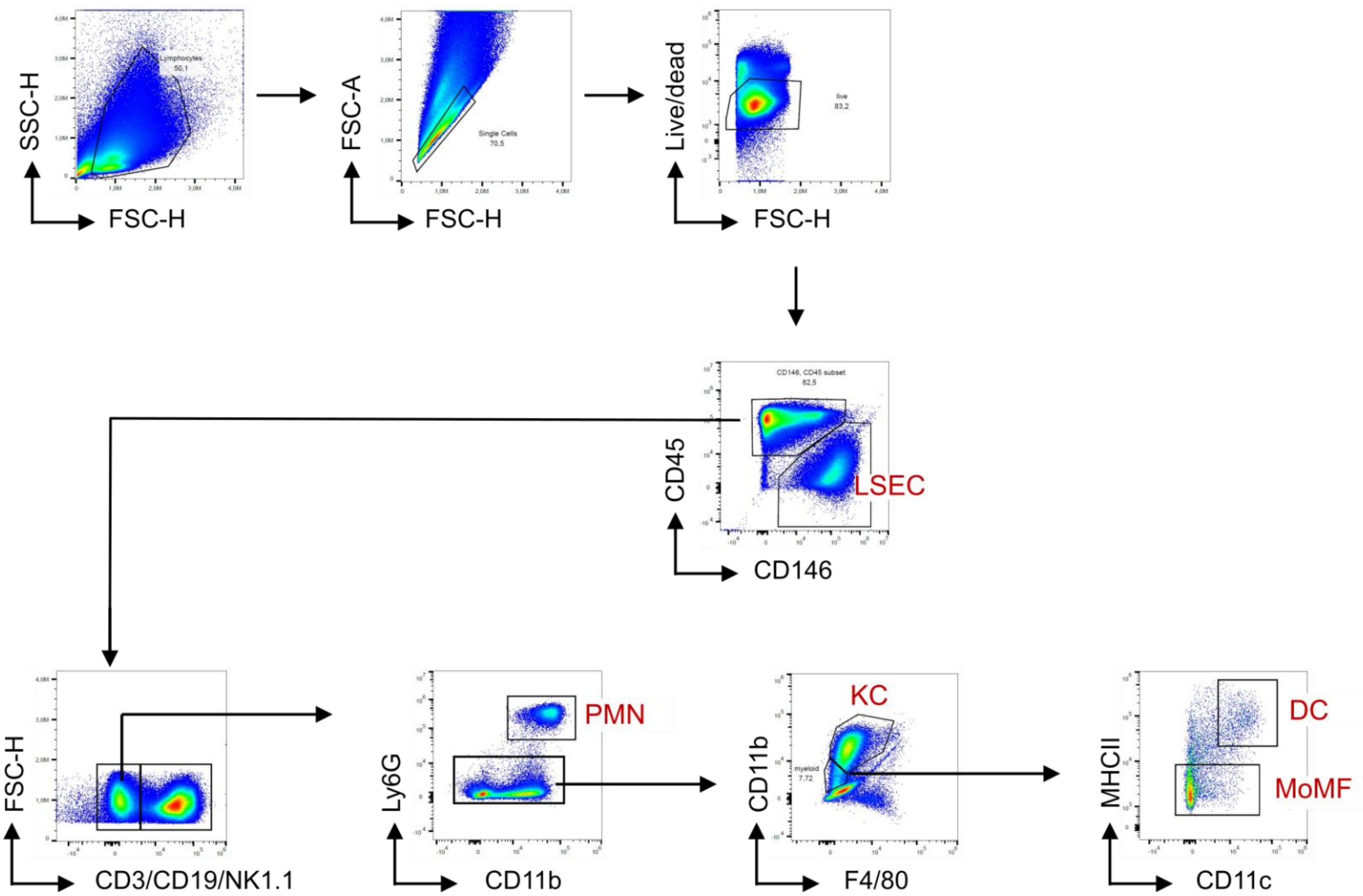

<u>Figure S1:</u> Strategy used in flow cytometry analyses to gate on the LSECs or on the other NPLC liver cells.

Gating strategy that we have used in our flow cytometry analyses to identify LSECs and to distinguish these cells from the remaining non-parenchymal liver cells (NPLC).
